# Supplementary material for: Understanding the effect of spatially separated Cu and acid sites in zeolite catalysts on oxidation of methane
Source: Nat Commun. 2024 Mar 28;15:2718. doi: 10.1038/s41467-024-46924-2 (PMC10978981; doi:10.1038/s41467-024-46924-2)
Supplement: Supplementary file 1 — Supplementary Information [file 41467_2024_46924_MOESM1_ESM.pdf]

*Supporting information*

# Understanding the effect of spatially separated Cu and acid sites in zeolite catalysts on oxidation of methane


Peipei Xiao [1], Yong Wang [1], Lizhuo Wang [2], Hiroto Toyoda [1], Kengo Nakamura [1], Samya Bekhti [1], Yao Lu [1], Jun Huang [2], Hermann Gies [1, 3], Toshiyuki Yokoi [1,4]*

[1] *Institute of Innovative Research, Tokyo Institute of Technology, 4259 Nagatsuta, Midori-ku, Yokohama 226-8503, Japan*

[2] *School of Chemical and Biomolecular Engineering, The University of Sydney, Sydney, New South Wales 2006, Australia*

[3] *Institute of Geology, Mineralogy und Geophysics, Ruhr-University Bochum, Bochum 44780, Germany*

[4] *iPEACE223 Inc. Konwa Building, 1-12-22 Tsukiji, Chuo-ku, Tokyo, 104-0045, Japan*

## Supplementary Methods

### Material Information

AEI zeolite was obtained from BASF SE, which was prepared based on the patent.[1]

ZSM-5 zeolite (JRC-Z5-30NH4) was provided by Mizusawa Industrial Chemicals, Ltd. to Japan Reference Catalyst (JRC).[2]

Beta zeolite (CP814E*) was purchased from Zeolyst International.[3]

### Catalyst preparation

**Cu/BEA.** 1Cu/BEA and 5Cu/BEA zeolite catalysts were prepared by using Beta (Zeolyst, CP814E*, $NH_4^+$ form) as the parent zeolite to ion exchange with 1 and 5 mmol/L $Cu(NO_3)_2$ (FUJIFILM, Wako Special Grade) solutions, respectively, at 80 ºC stirring for 24 h (Solid/Liquid ratio was 1g/100 ml). The solid products were washed, dried at 100 ºC overnight, and calcined at 550 °C for 5 h in air. The obtained samples were denoted as 1Cu/BEA and 5Cu/BEA, where 1 and 5 meant the $Cu(NO_3)_2$ concentration.

### Catalyst test in Methanol to hydrocarbons (MTH) reaction

The MTH reaction was performed using a fixed-bed reactor connected to an online gas-chromatograph (GC-2014, Shimadzu) equipped with a HP-PLOT/Q capillary column and a flame ionization detector. The 50/80 mesh zeolite pellets without a binder were placed in a 6 mm quartz tubular flow reactor. The pretreatment was conducted at 500 °C for 30 min under Ar (20 $mL\cdot min^{-1}$). After the pretreatment, the reactor was cooled to 350 °C and the MTH reaction was started. The pressure of methanol was set at 5 kPa with Ar gas as the carrier; the weight-to-feed ratio (W/F) for methanol was 68 $g\cdot h\cdot mol^{-1}$. The product stream was analyzed using a system that automatically injected the product into a gas chromatograph connected directly to the outlet of the reactor via a heated transfer line.

The conversion and selectivity were calculated as:

$$Conversion\ of\ methanol\ [\%] = 1 - \frac{\text{Amount of methanol (in reacted gas)}}{\text{Amount of detected compounds (in reacted gas)}} \times 100 \qquad (1)$$

$$Product\ selectivity\ [\%] = \frac{\text{Amount of the target product}}{\text{Amount of detected products (in reacted gas)}} \times 100 \qquad (2)$$

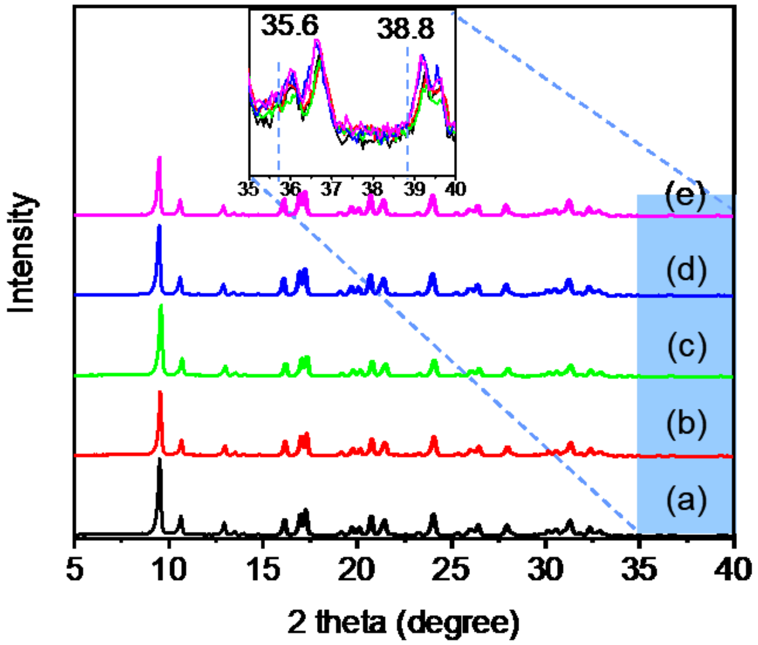


**Supplementary Figure 1. XRD patterns of *x*Cu/AEI zeolite catalysts.** (a) H-AEI, (b) 1Cu/AEI, (c) 5Cu/AEI, (d) 50Cu/AEI, (e) 500Cu/AEI zeolite catalysts.

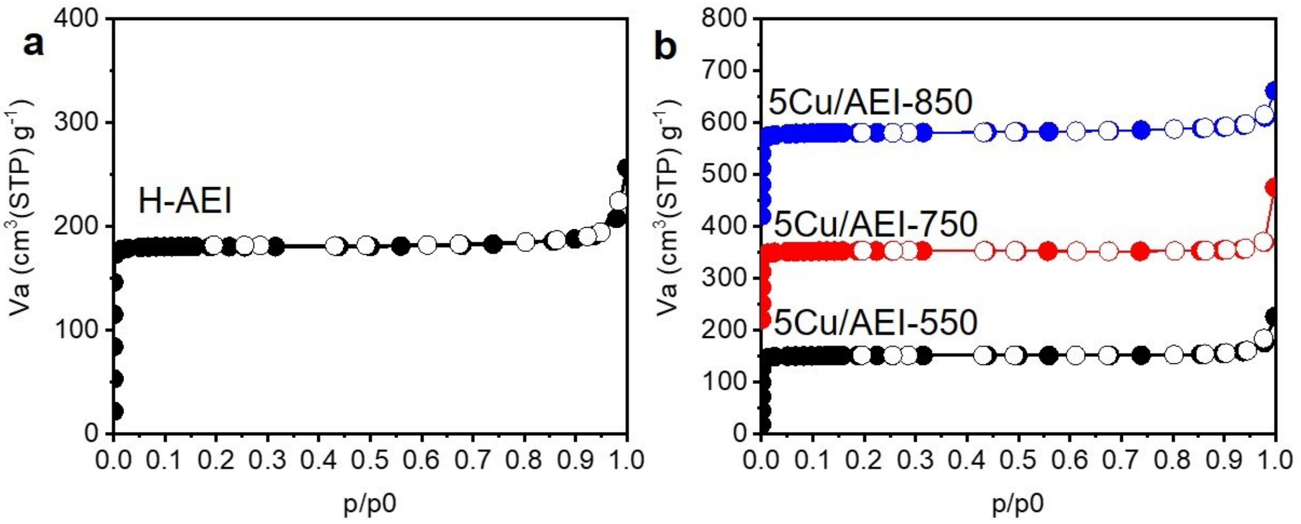


**Supplementary Figure 2. $N_2$ adsorption and desorption curves.** (a) H-AEI and (b) 5Cu/AEI-*t* zeolite catalysts.

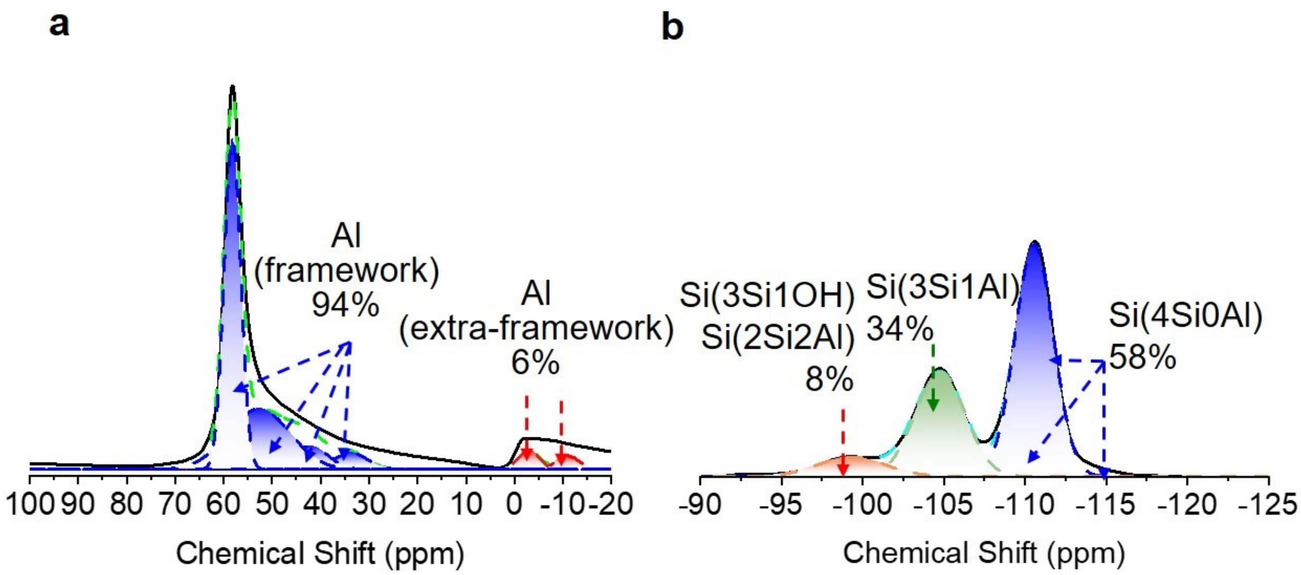


**Supplementary Figure 3. NMR result for H-AEI zeolite.** (a) Deconvolution of $^{27}$Al MAS NMR and (b) deconvolution of $^{29}$Si MAS NMR.

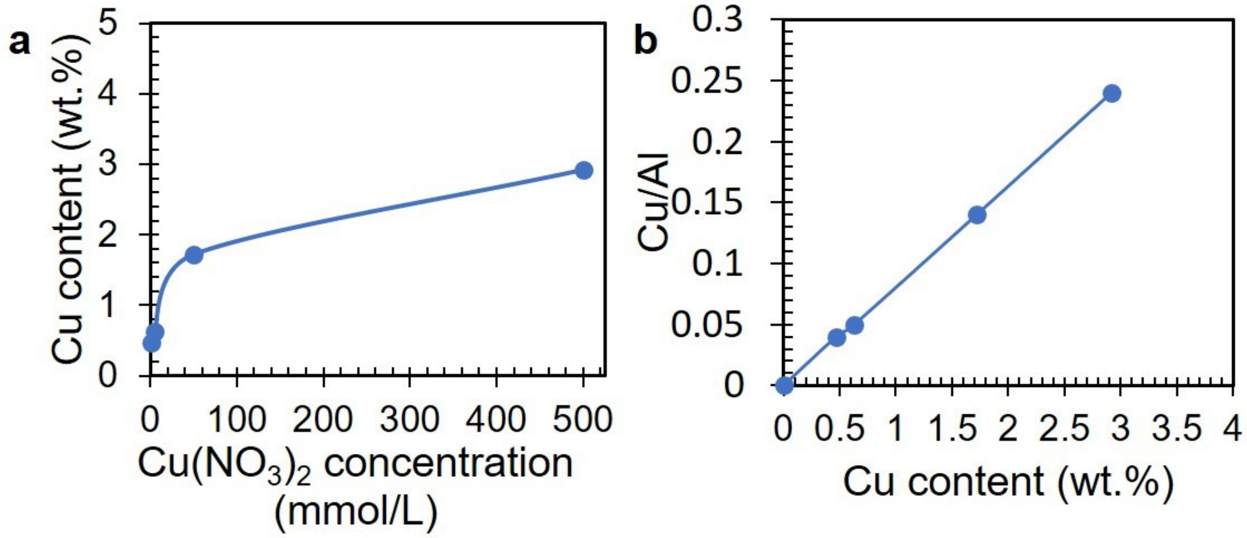


**Supplementary Figure 4. Relation between input $Cu(NO_3)_2$ concentration and output Cu content.** (a) $Cu(NO_3)_2$ concentration in the solution as a function of Cu content in the Cu/AEI zeolite catalysts. (b) Cu content as a function of Cu/Al in the Cu/AEI zeolite catalysts.

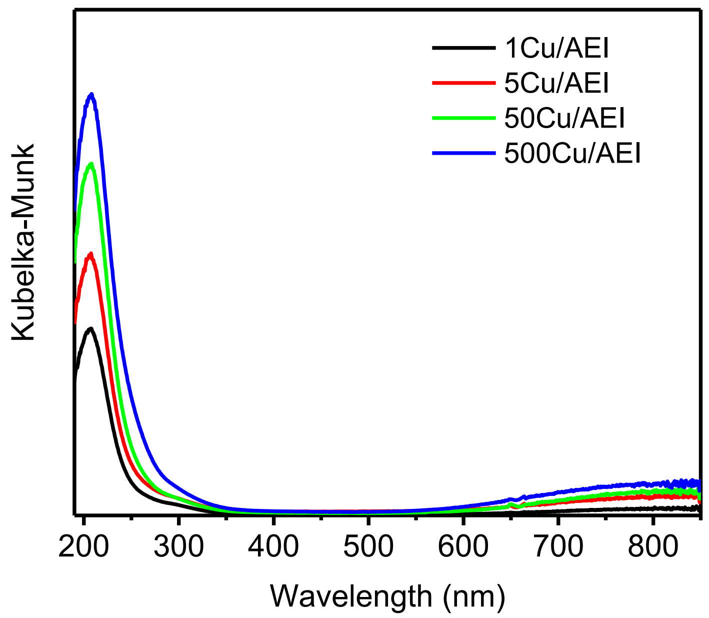


**Supplementary Figure 5. UV-vis spectra of the *x*Cu/AEI zeolite catalysts.** Measured at atmospheric pressure and atmospheric temperature without pretreatment.

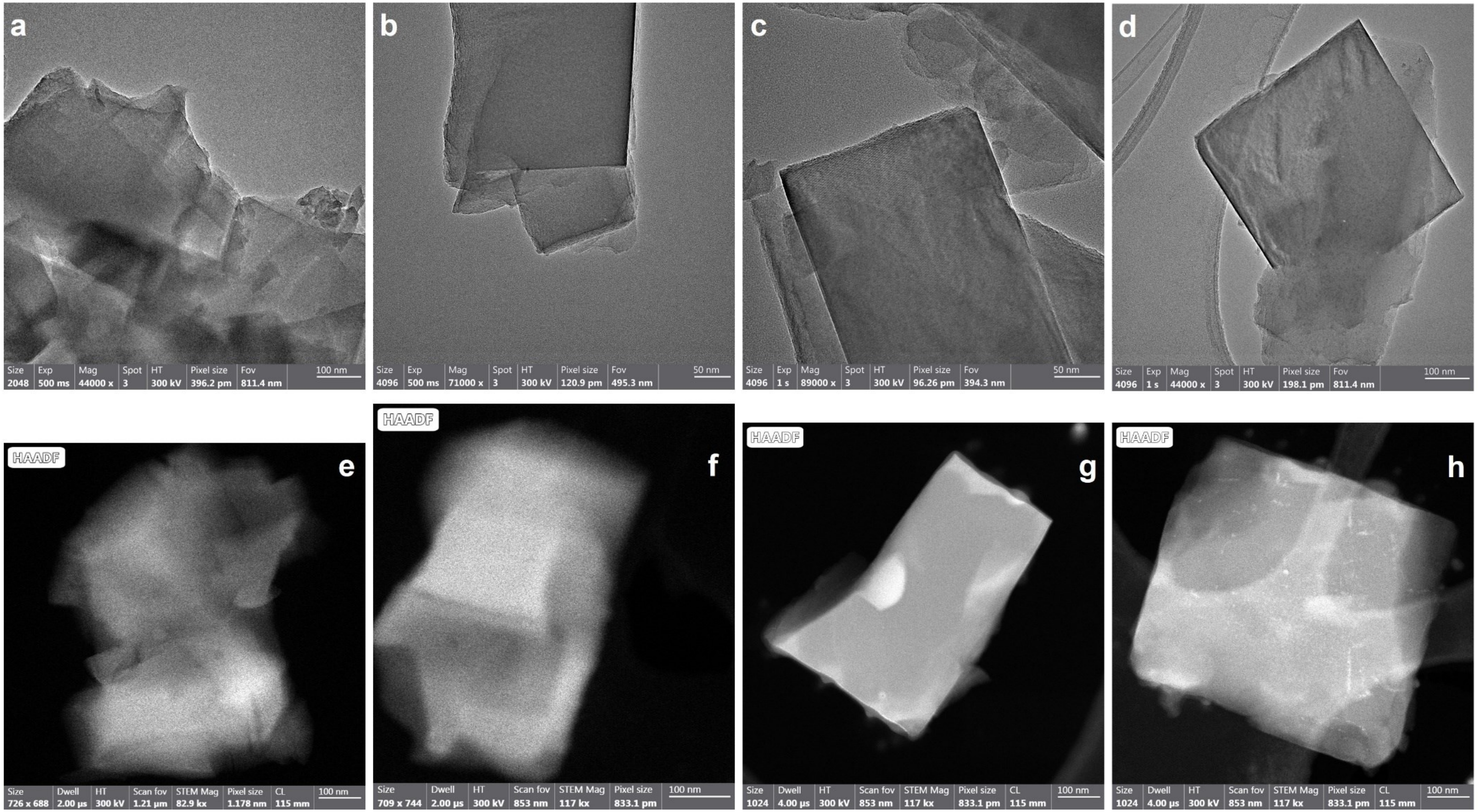


**Supplementary Figure 6. HRTEM and HAADF-STEM images**. (a and e) 1Cu/AEI, (b and f) 5Cu/AEI, (c and g) 50Cu/AEI, (d and h) 500Cu/AEI zeolite catalysts.

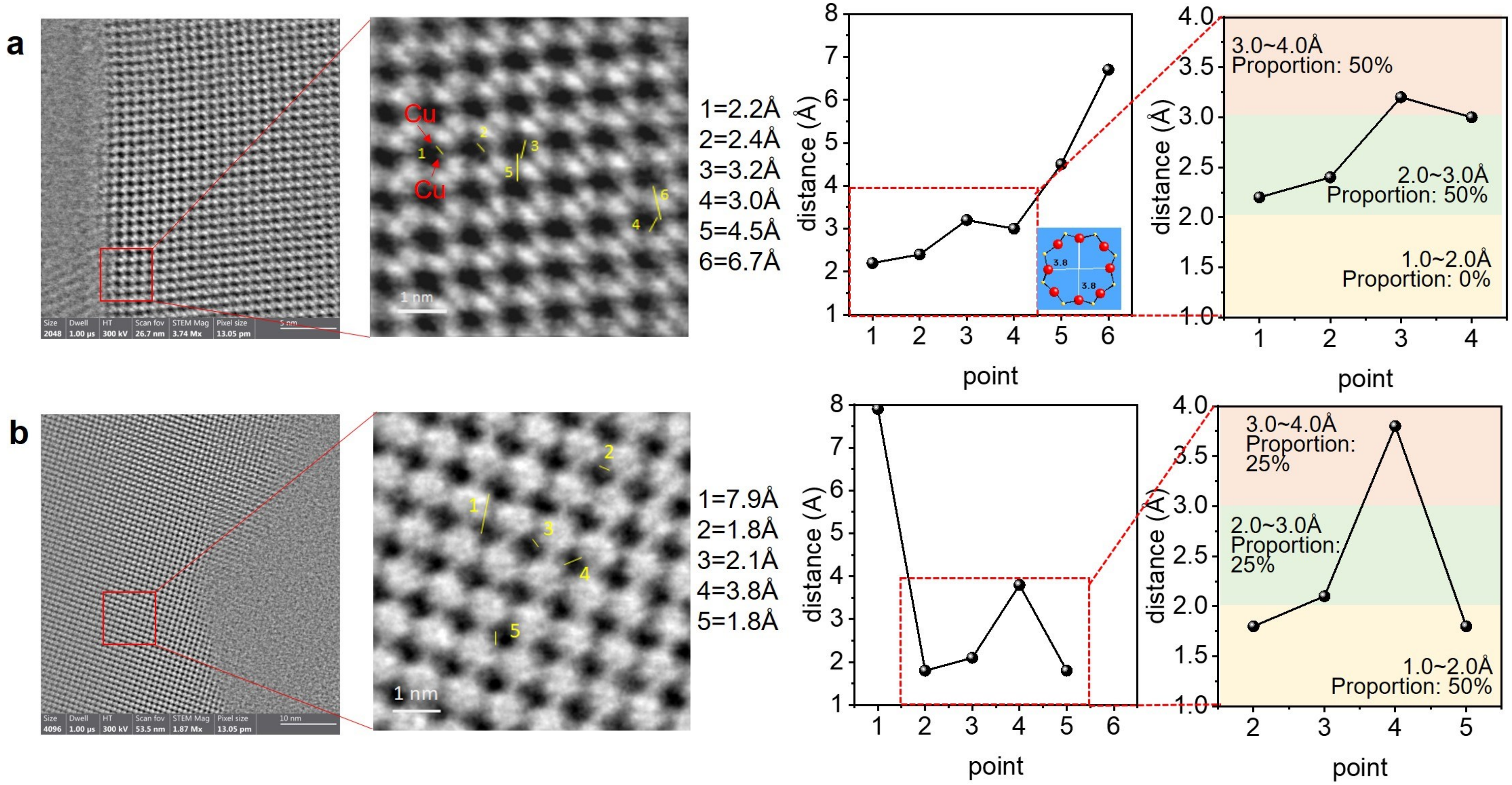


**Supplementary Figure 7. Representative HAADF-STEM images to partially reveal the distance between Cu and Cu.** (a) 1Cu/AEI and (b) 500Cu/AEI zeolite catalysts.

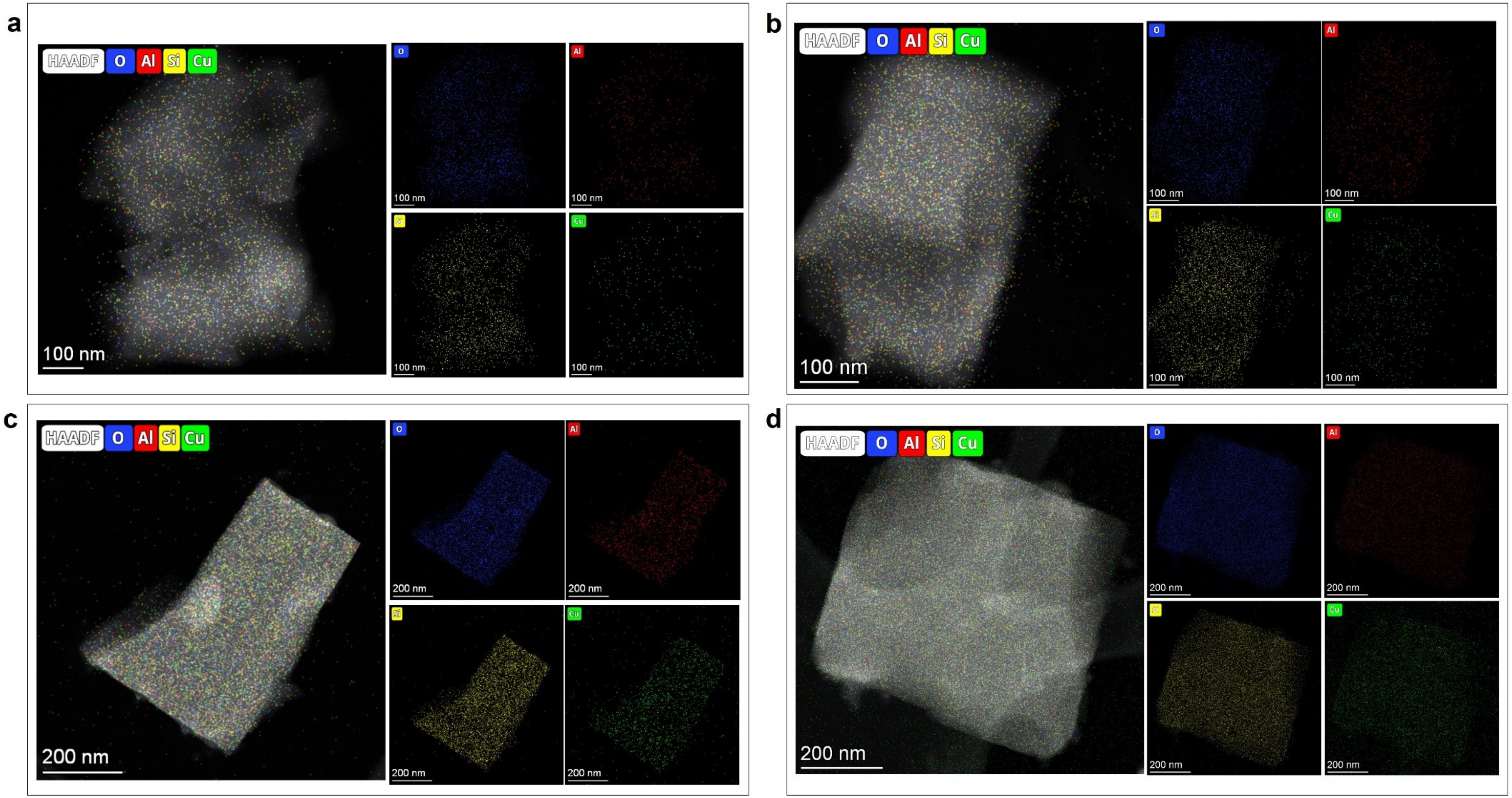


**Supplementary Figure 8. EDS elemental mapping images of overlay of O, Al, Si, Cu, and independent O, Al, Si, Cu.** (a) 1Cu/AEI, (b) 5Cu/AEI, (c) 50Cu/AEI, (d) 500Cu/AEI zeolite catalysts.

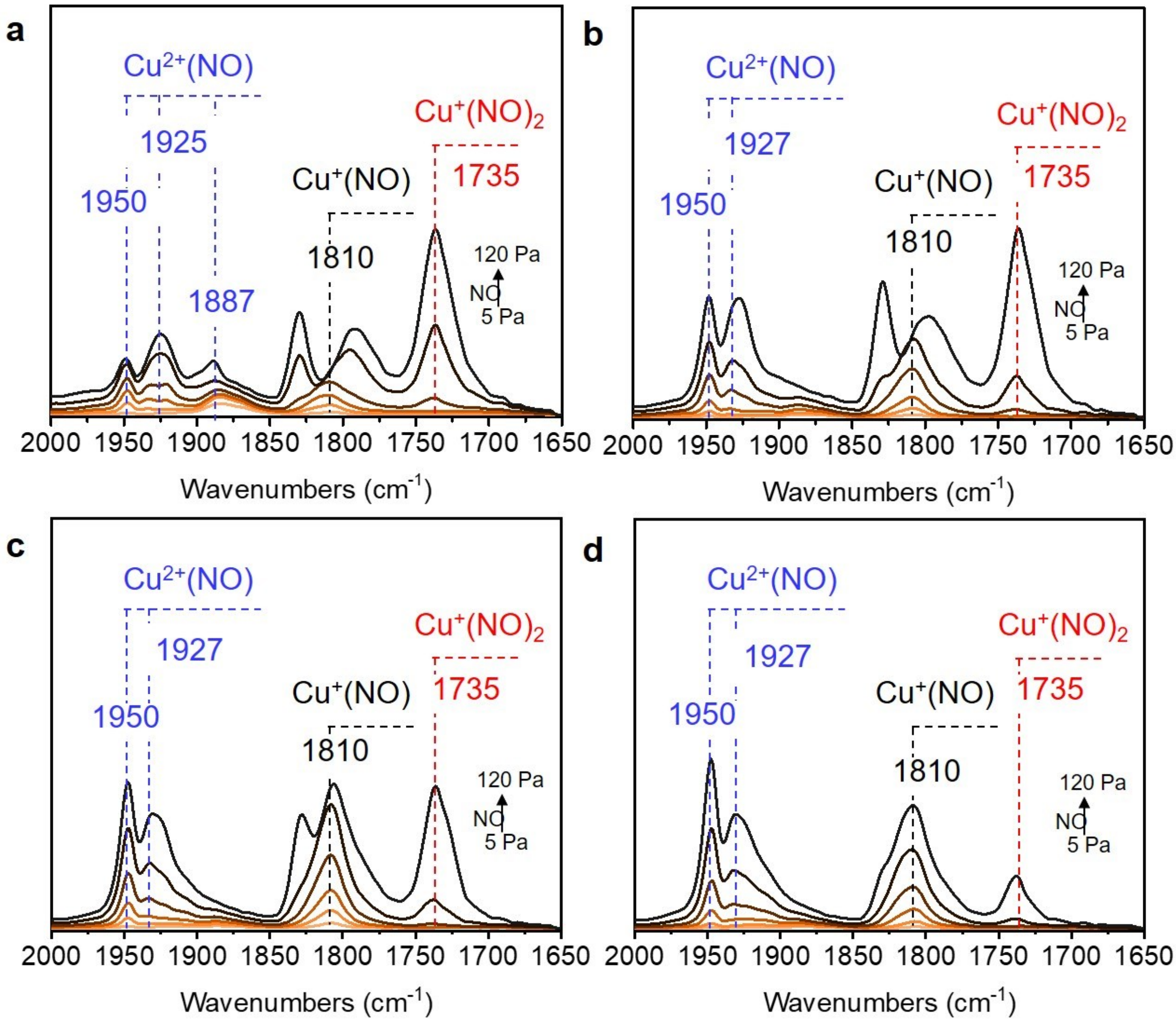


**Supplementary Figure 9. FTIR spectra of adsorbed NO (5-120 Pa) at -120 $^{o}$C over *x*Cu/AEI zeolites.** (a) 1Cu/AEI, (b) 5Cu/AEI, (c) 50Cu/AEI, (d) 500Cu/AEI zeolite catalysts.

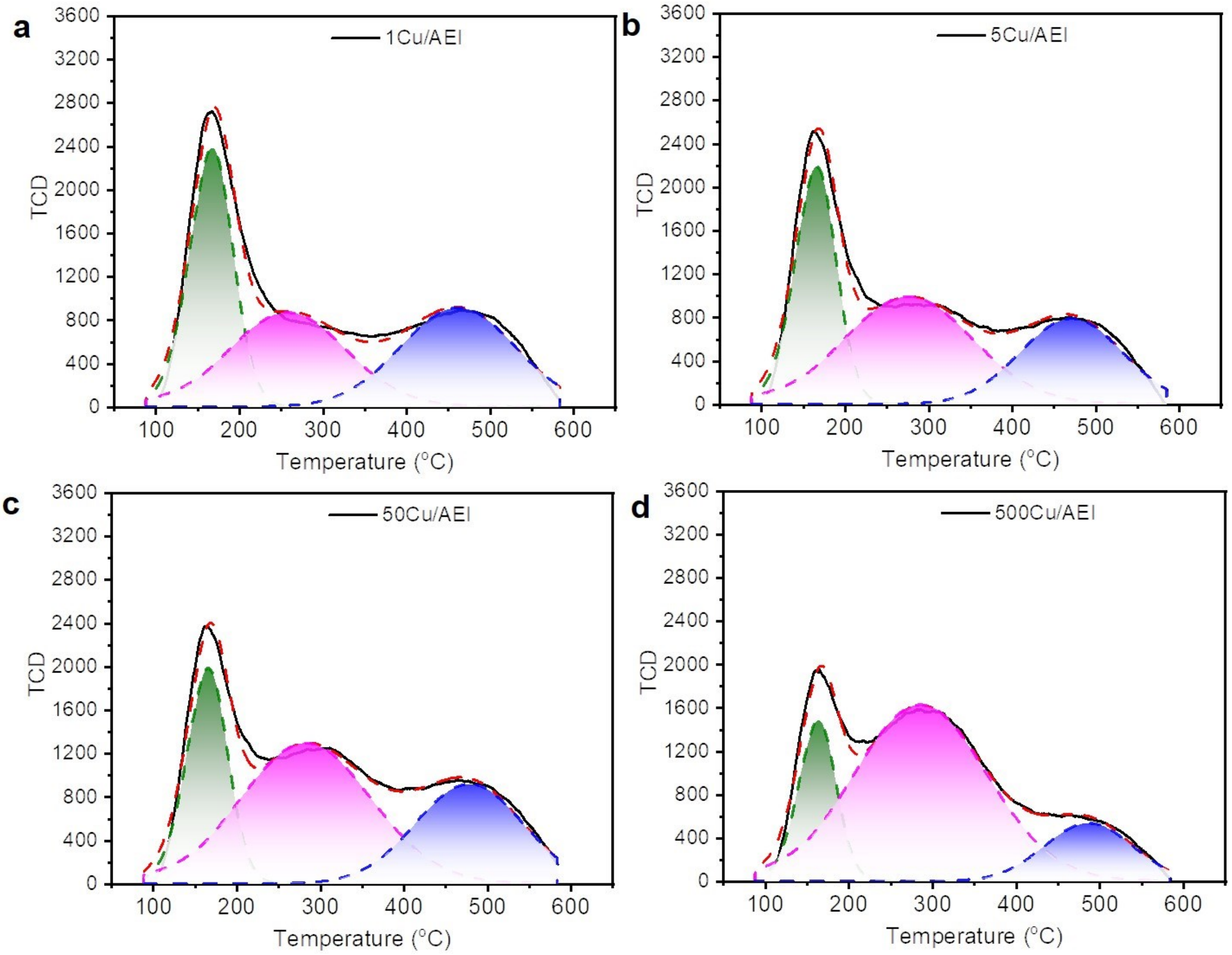


**Supplementary Figure 10. $NH_3$-TPD curves of *x*Cu/AEI zeolites.** (a) 1Cu/AEI, (b) 5Cu/AEI, (c) 50Cu/AEI, (d) 500Cu/AEI zeolite catalysts.

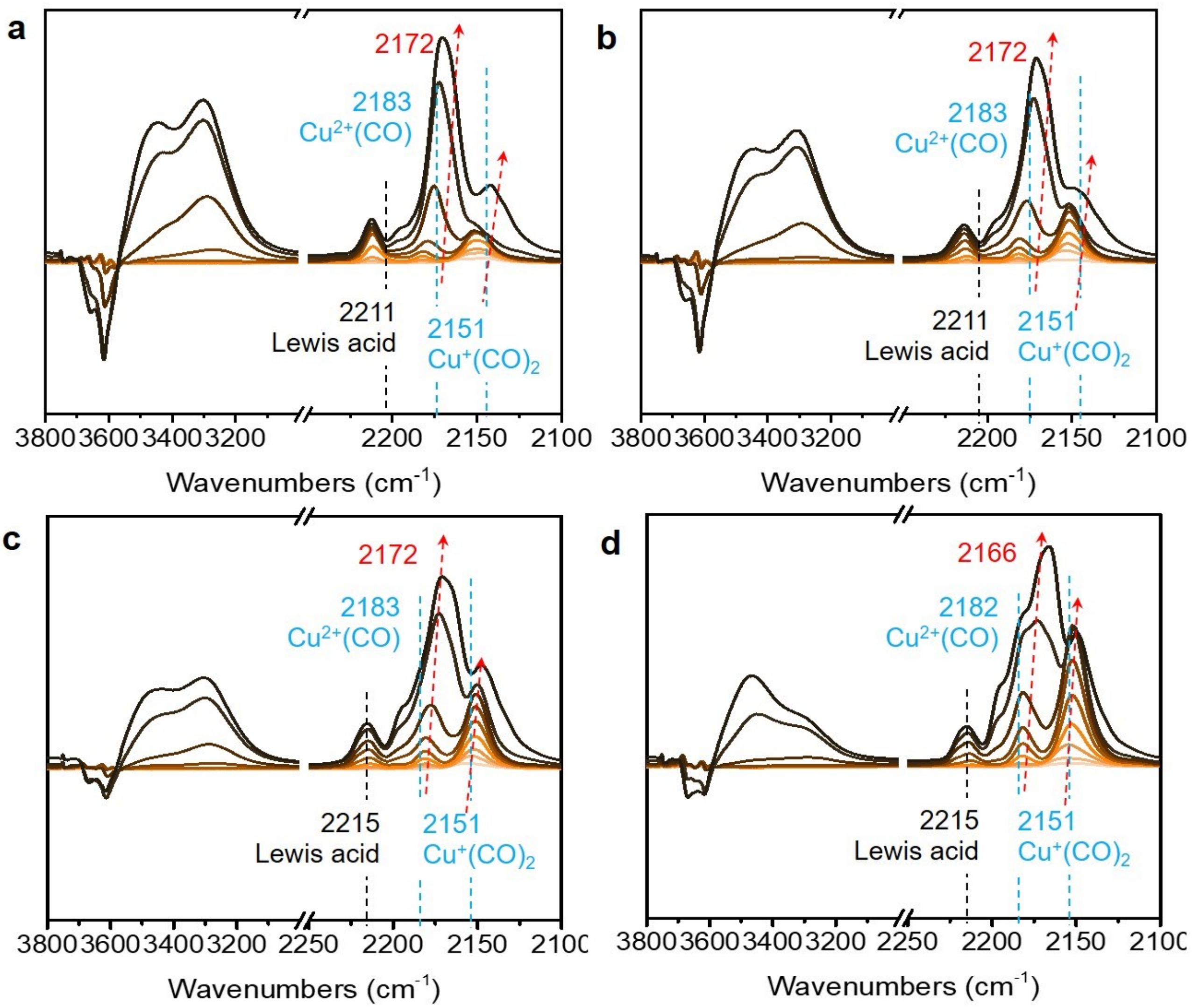


**Supplementary Figure 11. FTIR spectra of adsorbed CO (5-1000 Pa) at -120 °C over *x*Cu/AEI zeolites.** (a) 1Cu/AEI, (b) 5Cu/AEI, (c) 50Cu/AEI, (d) 500Cu/AEI zeolite catalysts.

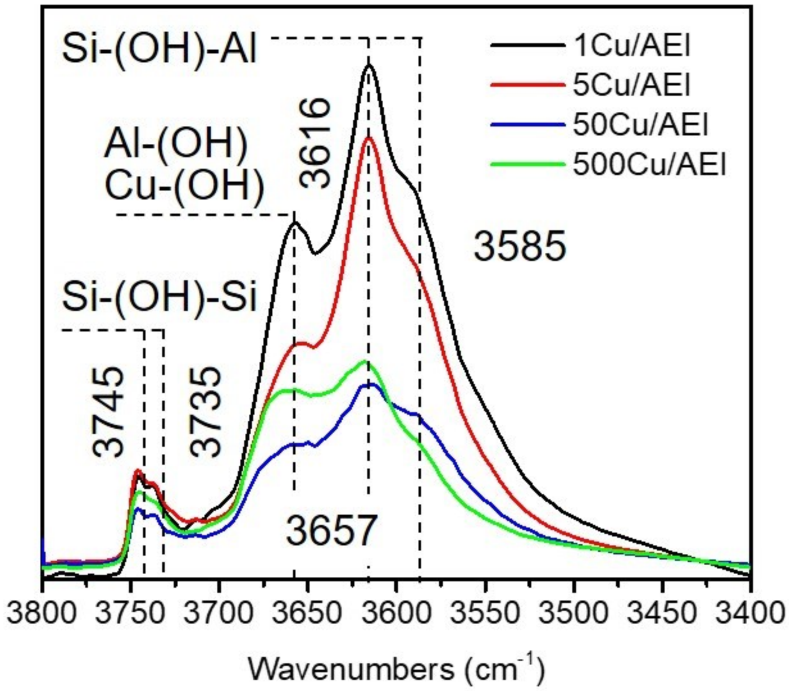


**Supplementary Figure 12. FTIR spectra in the OH region of *x*Cu/AEI samples.** Collected at -120 ºC after activation at 500 ºC in vacuum for 1 h.

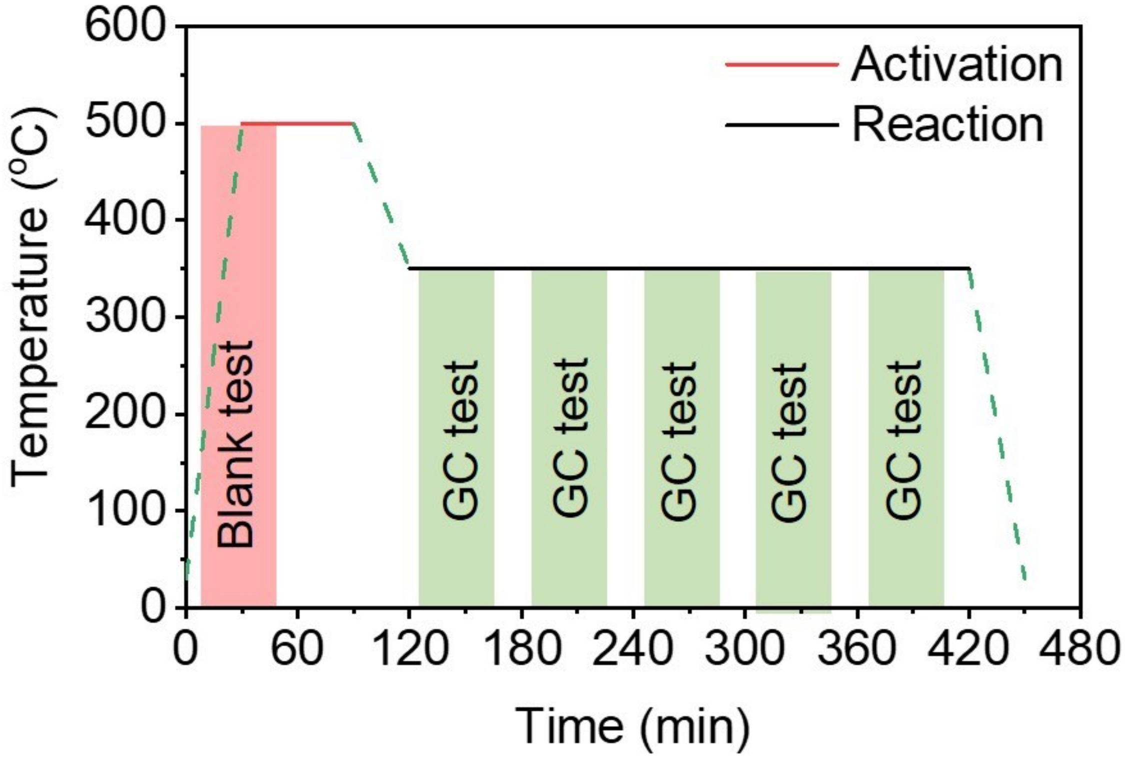


**Supplementary Figure 13. Temperature program of the continuous oxidation of methane.** Reaction at 350 ºC after activation at 500 ºC for 1h.

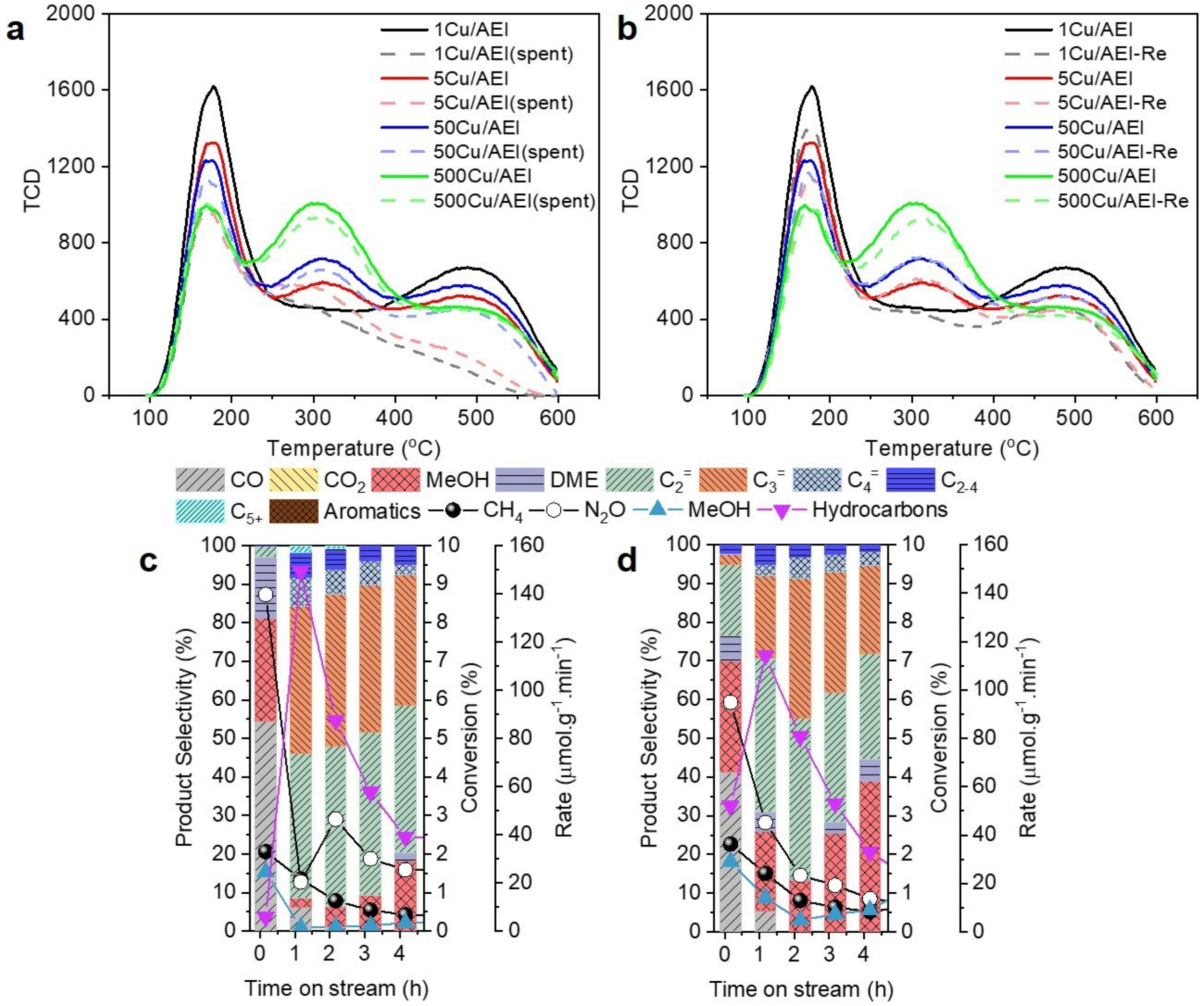


**Supplementary Figure 14. Compare the properties of the fresh, spent, and regenerated *x*Cu/AEI zeolites.** (a) Compare the $NH_3$-TPD patterns of fresh (*x*Cu/AEI) and spent (*x*Cu/AEI(spent)) zeolite catalysts. (b) Compare the $NH_3$-TPD patterns of fresh (*x*Cu/AEI) and spent samples after regeneration by calcination at 550 °C for 5 h (*x*Cu/AEI-Re). Note that these samples were measured by BELCAT-A with BELMass. Compare the reaction performance of (c) fresh 1Cu/AEI zeolite and (d) regenerated 1Cu/AEI zeolite by calcination at 550 °C for 5 h. Reaction conditions: 100 mg catalyst, 350 °C, $CH_4/N_2O/H_2O/Ar$ = 10/10/2/3 ml·min$^{-1}$.

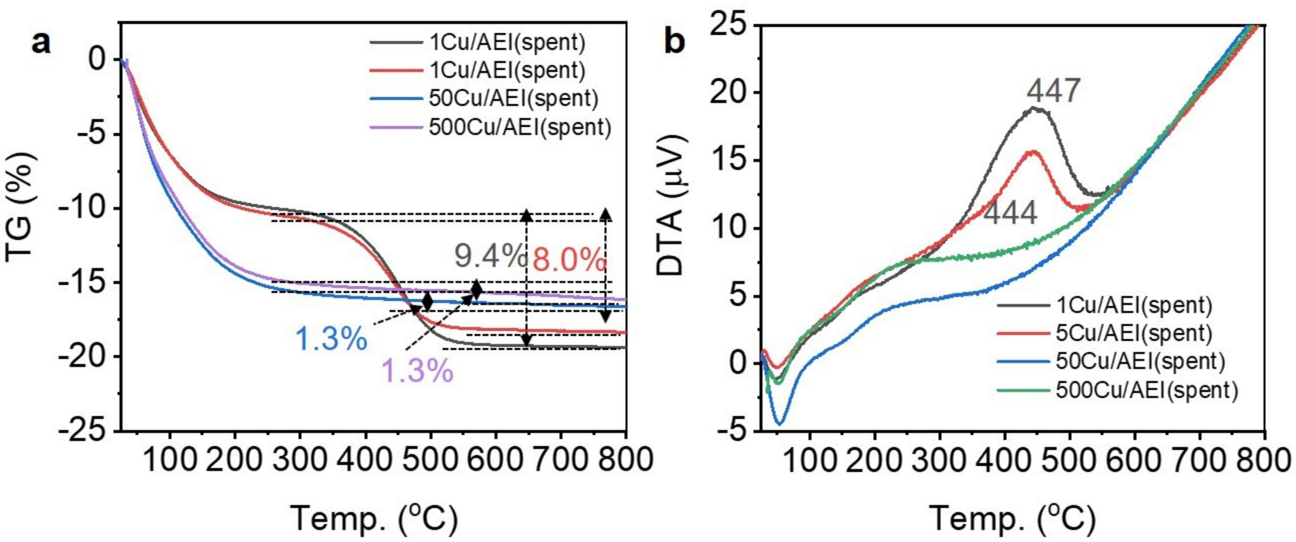


**Supplementary Figure 15. TG-DTA curves of the spent *x*Cu/AEI zeolites.** (a) TG analysis and (b) DTA curves.

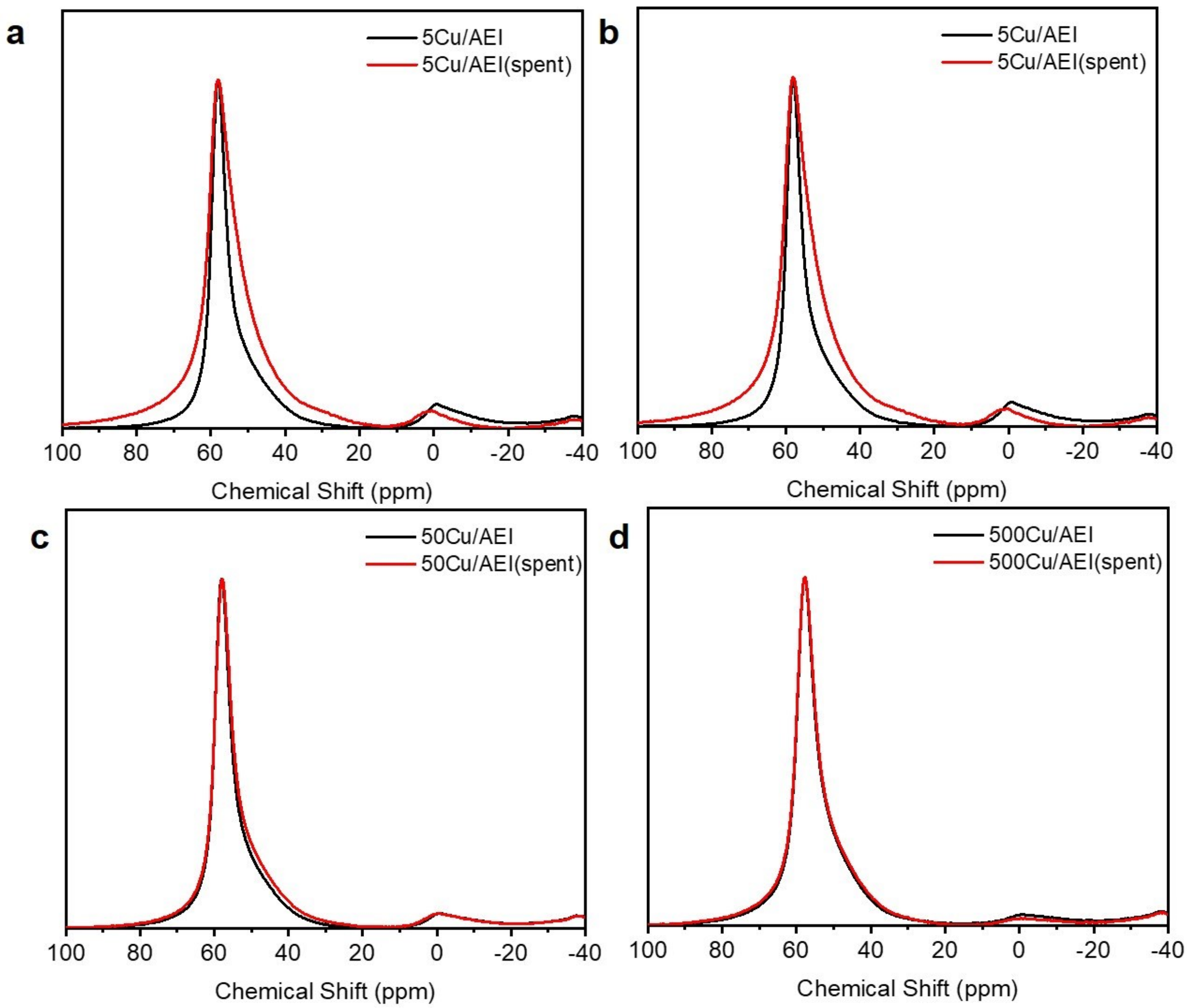


**Supplementary Figure 16. Compare the $^{27}$Al MAS NMR results of fresh and spent samples.** (a) 1Cu/AEI, (b) 5Cu/AEI, (c) 50Cu/AEI and (d) 500Cu/AEI zeolites.

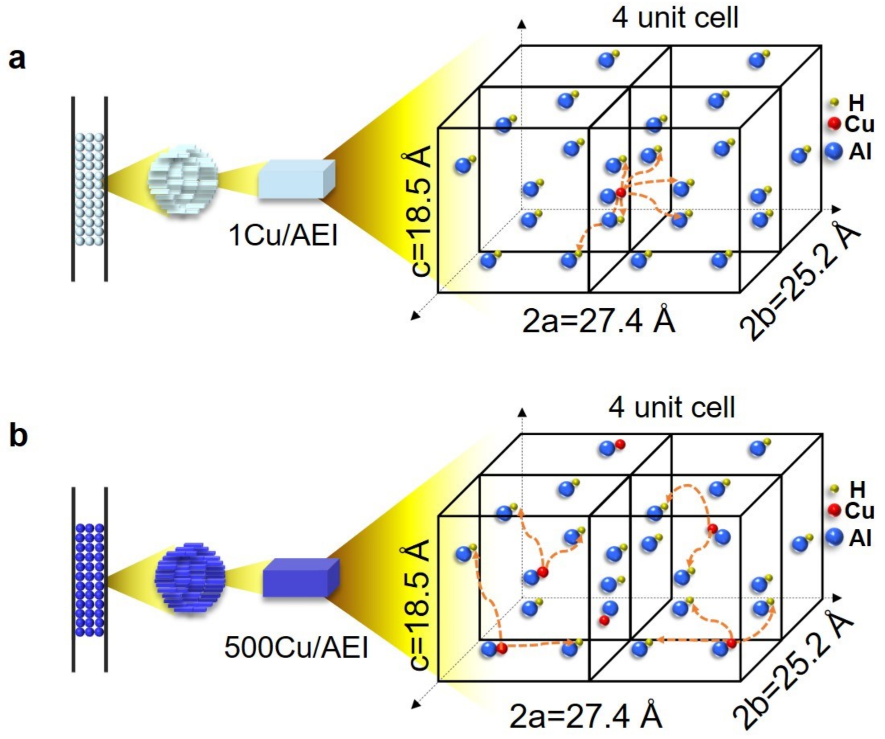


**Supplementary Figure 17. Schematic diagram of intimacy between Cu and acid sites.** (a) 1Cu/AEI and (b) 500Cu/AEI zeolite.

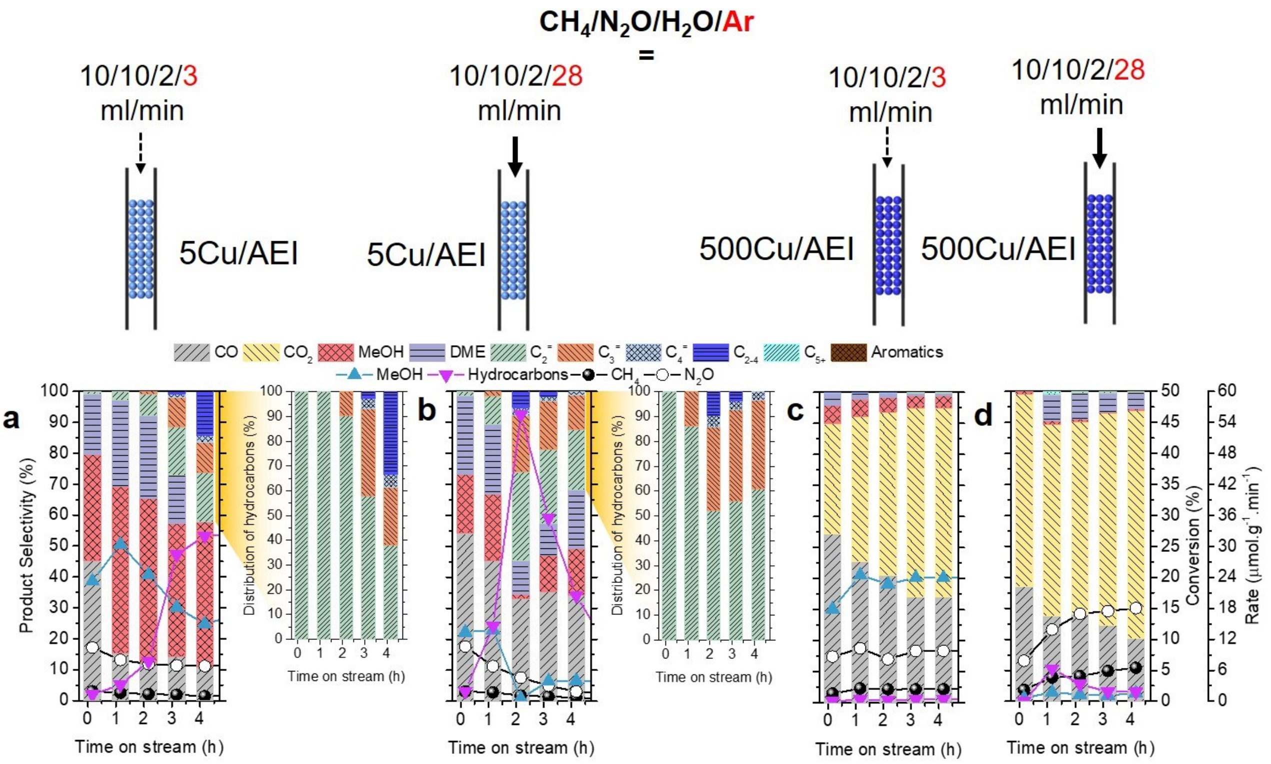


**Supplementary Figure 18. Effect of weight hour space velocity (WHSV) by adjusting the flow rate on reaction performance of direct oxidation of methane at 350 $^{o}$C.** (a) 100 mg 5Cu/AEI, $CH_4/N_2O/H_2O/Ar$=10/10/2/3 ml·min$^{-1}$, WHSV=15000 ml·g$^{-1}$·h$^{-1}$. (b) 100 mg 5Cu/AEI, $CH_4/N_2O/H_2O/Ar$=10/10/2/28 ml·min$^{-1}$, WHSV=30000 ml·g$^{-1}$·h$^{-1}$. (c) 100 mg 500Cu/AEI, $CH_4/N_2O/H_2O/Ar$=10/10/2/3 ml·min$^{-1}$, WHSV=15000 ml·g$^{-1}$·h$^{-1}$. (d) 100 mg 500Cu/AEI, $CH_4/N_2O/H_2O/Ar$=10/10/2/28 ml·min$^{-1}$, WHSV=30000 ml·g$^{-1}$·h$^{-1}$.

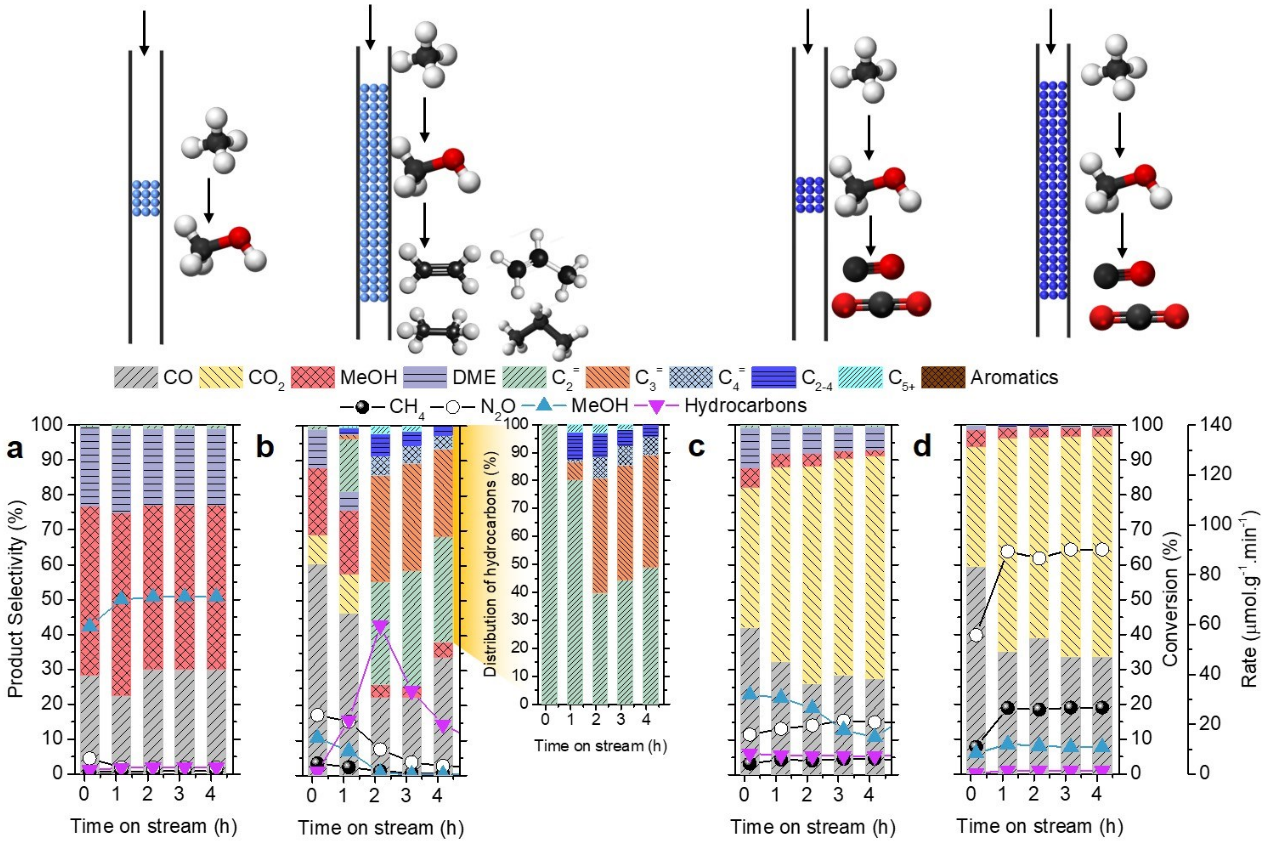


**Supplementary Figure 19. Effects of weight hour space velocity (WHSV) by adjusting catalyst amount on reaction performance of direct oxidation of methane at 350 ºC..** (a) 25 mg 5Cu/AEI (WHSV=60000 ml·g$^{-1}$·h$^{-1}$), (b) 200 mg 5Cu/AEI (WHSV=7500 ml·g$^{-1}$·h$^{-1}$), (c) 25 mg 500Cu/AEI (WHSV=60000 ml·g$^{-1}$·h$^{-1}$), and (d) 200 mg 500Cu/AEI (WHSV=7500 ml·g$^{-1}$·h$^{-1}$).

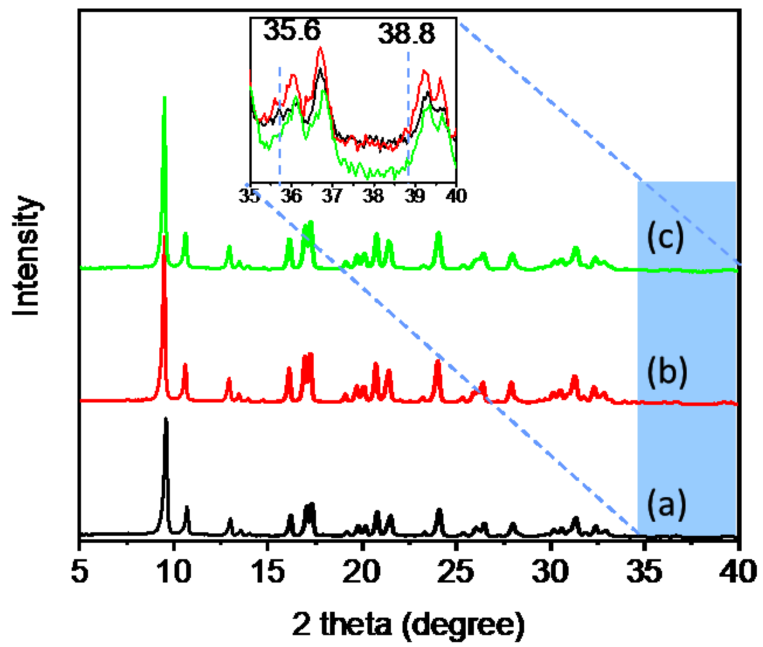


**Supplementary Figure 20. XRD patterns of 5Cu/AEI-*t* zeolite catalysts.** (a) 5Cu/AEI-550, (b) 5Cu/AEI-750, (c) 5Cu/AEI-850.

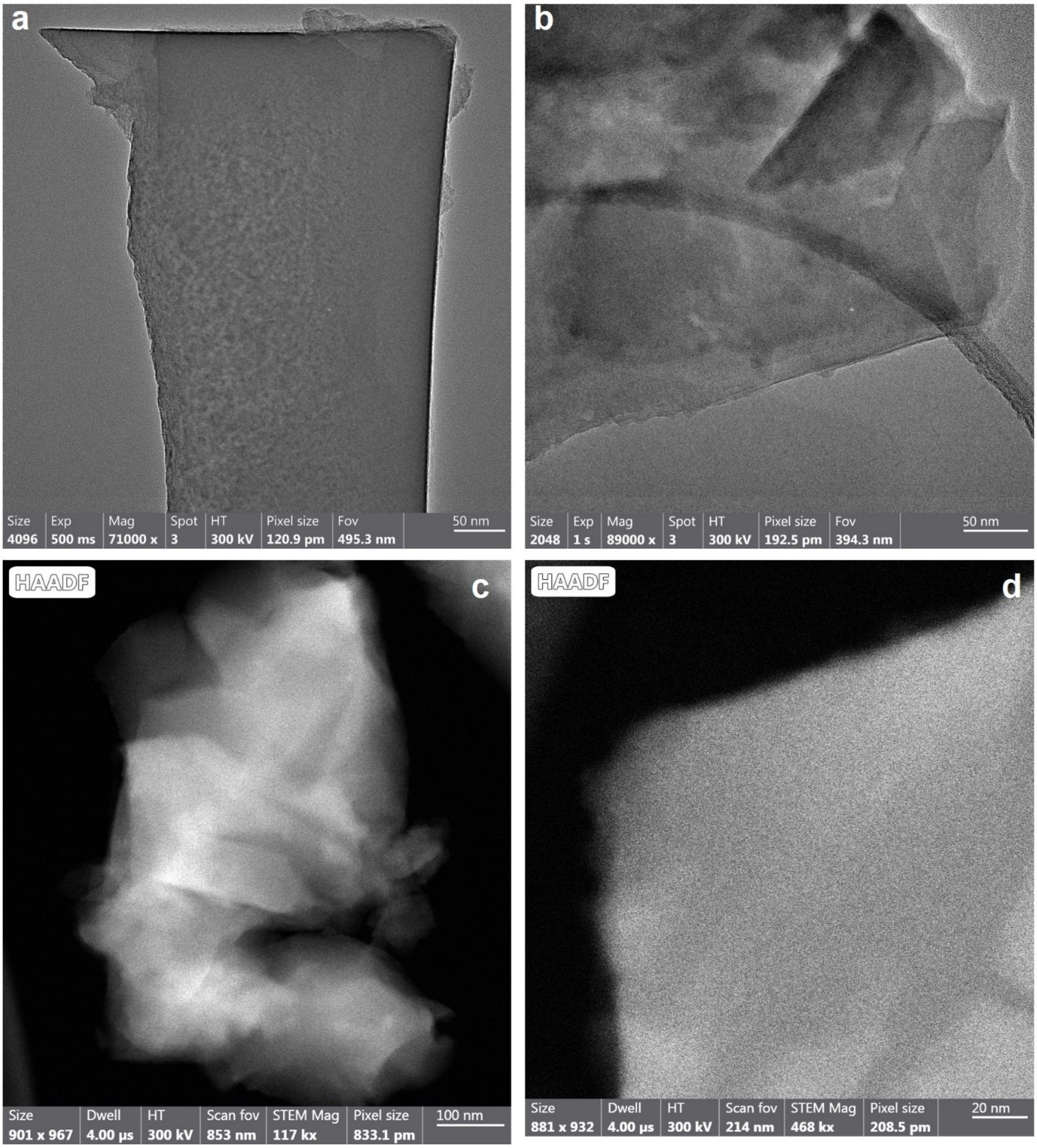


**Supplementary Figure 21. HRTEM and HAADF-STEM images.** (a and c) 5Cu/AEI-750 and (b and d) 5Cu/AEI-850 zeolite catalysts.

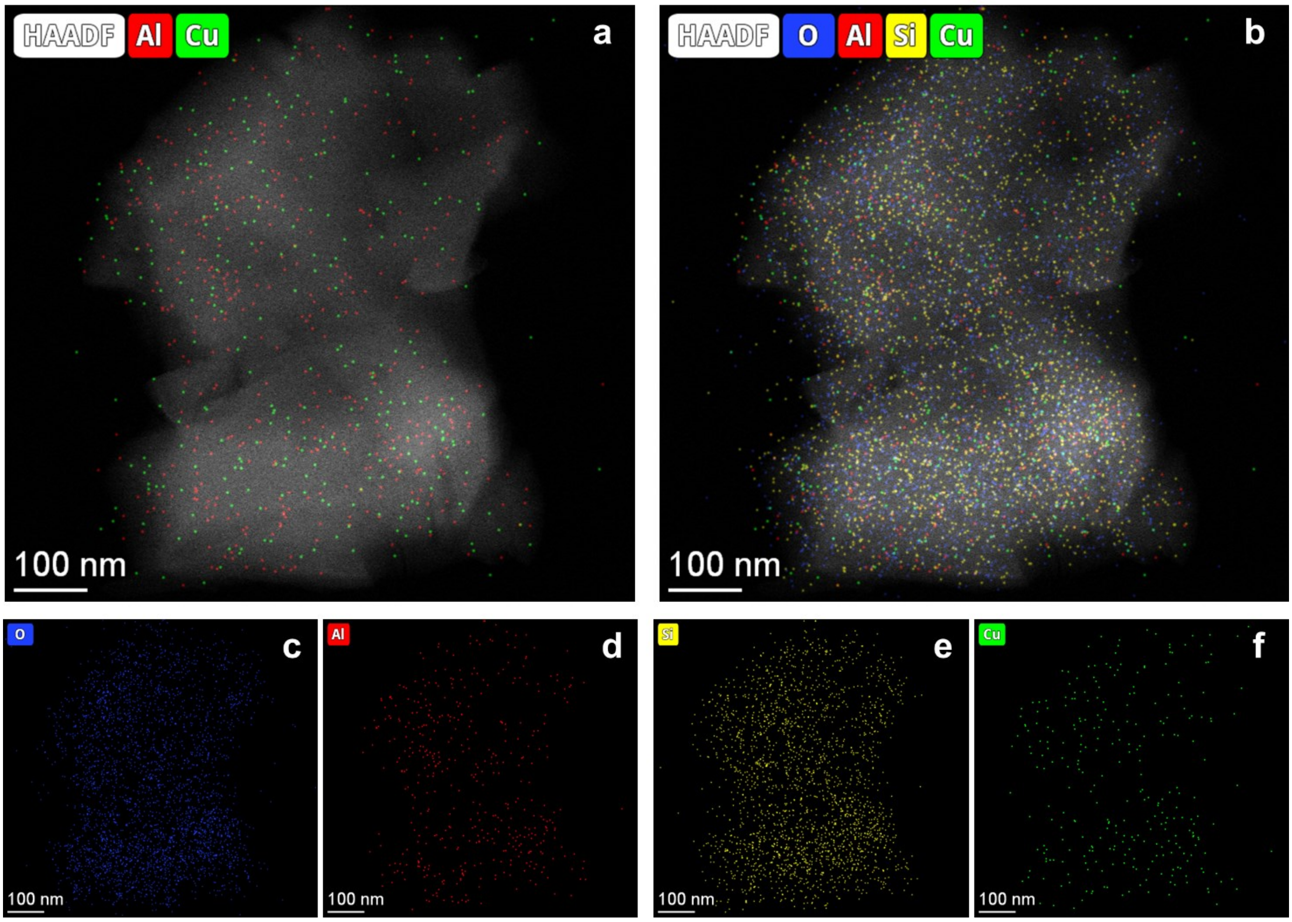


**Supplementary Figure 22. EDS elemental mapping images for 5Cu/AEI-750 zeolite catalyst.** (a) overlay Cu and Al, (b) overlay all the elements, and independent (c) O, (d) Al, (e) Si, (f) Cu.

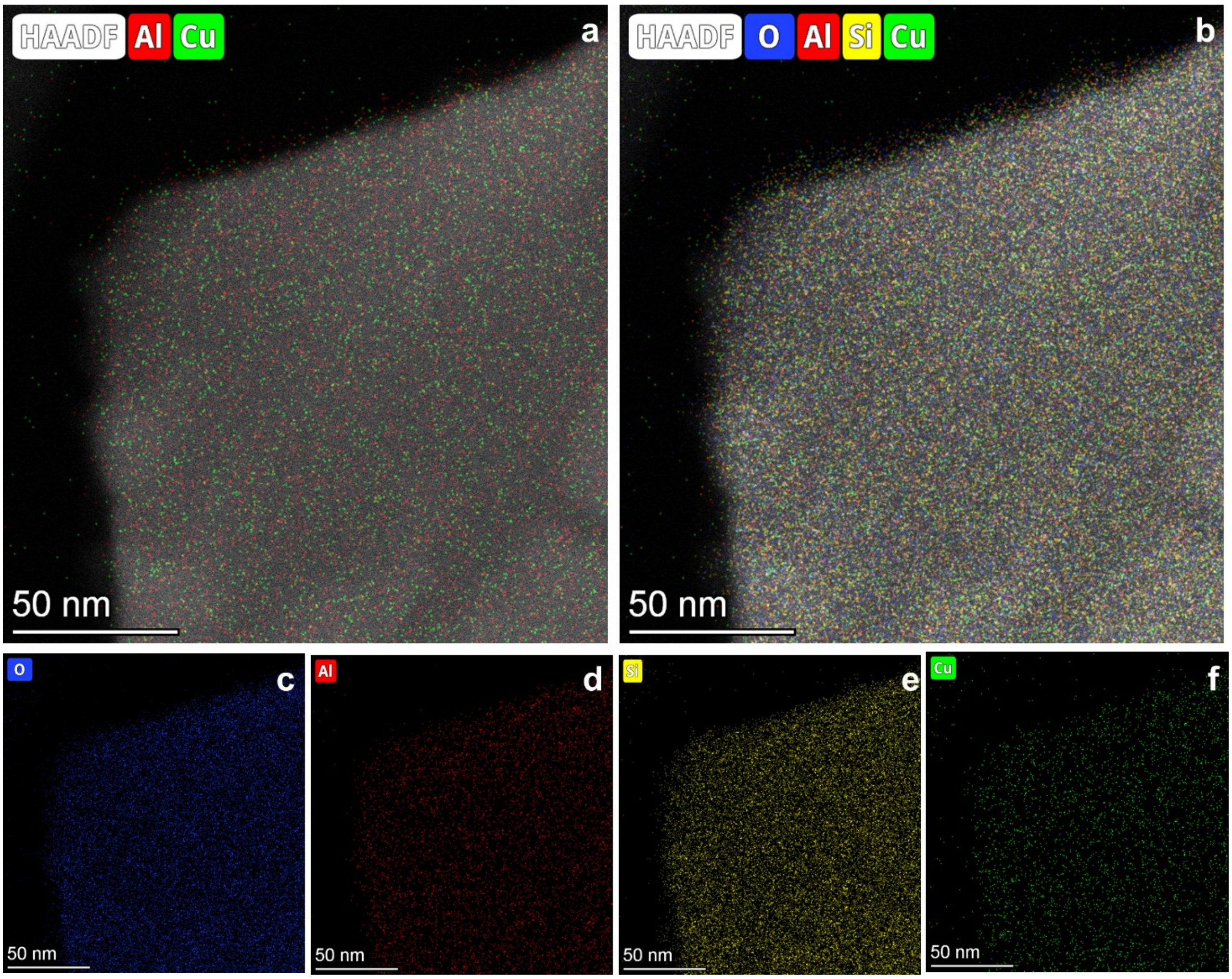


**Supplementary Figure 23. EDS elemental mapping images for 5Cu/AEI-850 zeolite catalyst.** (a) overlay Cu and Al, (b) overlay all the elements, and independent (c) O, (d) Al, (e) Si, (f) Cu.

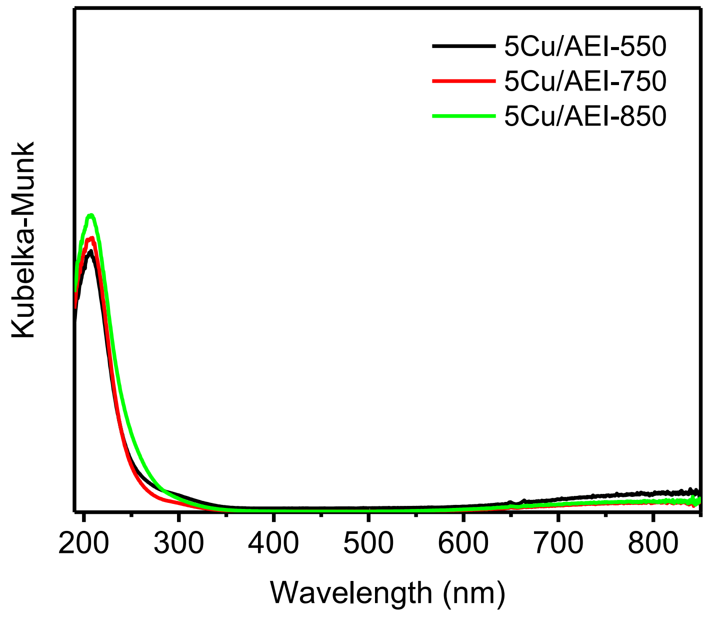


**Supplementary Figure 24. UV-vis spectra of 5Cu/AEI-*t* zeolite catalysts.** Measured at atmospheric pressure and atmospheric temperature without pretreatment.

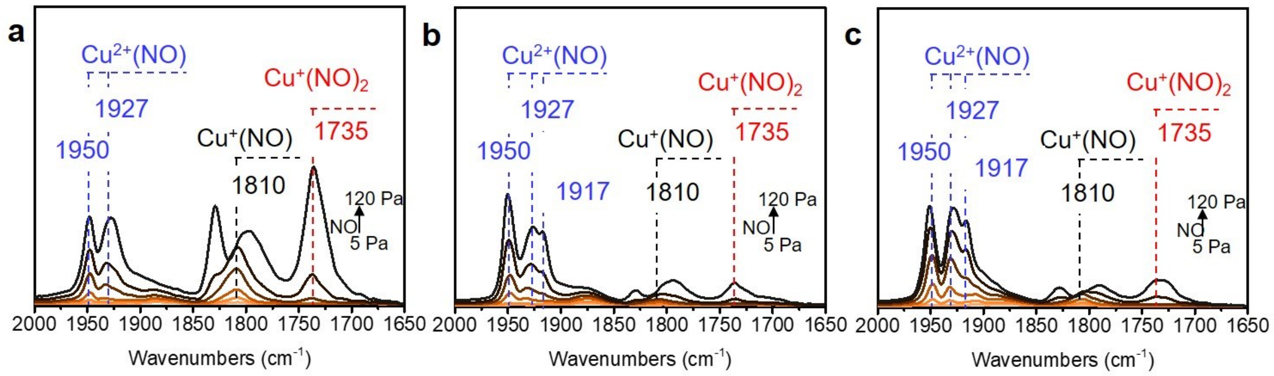


**Supplementary Figure 25. FTIR spectra of adsorbed NO (5-120 Pa) at -120 $^{o}$C after evacuation at 500 $^{o}$C for 1 h.** (a) 5Cu/AEI-550, (b) 5Cu/AEI-750, (c) 5Cu/AEI-850 zeolite catalysts.

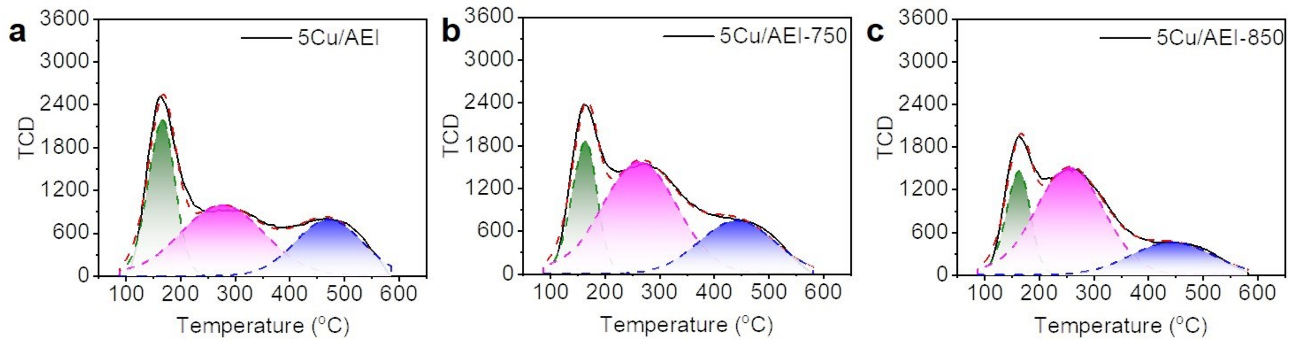


**Supplementary Figure 26. $NH_3$-TPD curves.** (a) 5Cu/AEI-550, (b) 5Cu/AEI-750, (c) 5Cu/AEI-850 zeolite catalysts.

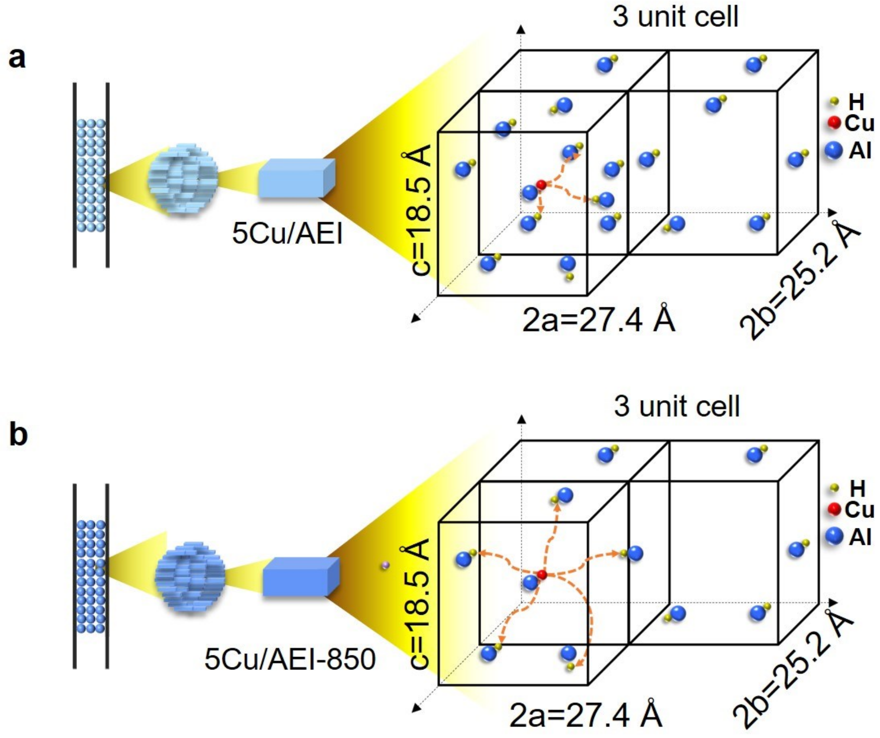


**Supplementary Figure 27. Schematic diagram of intimacy between Cu and acid sites.** (a) 5Cu/AEI (*i.e.* 5Cu/AEI-550) and (b) 5Cu/AEI-850 zeolite catalysts.

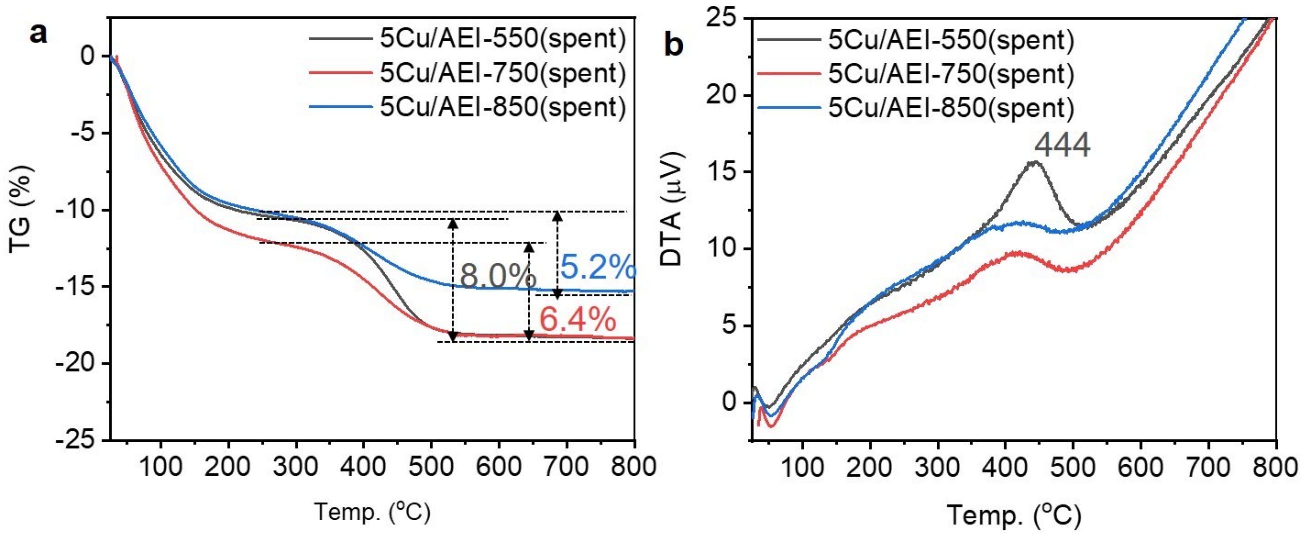


**Supplementary Figure 28. TG-DTA curves of the spent 5Cu/AEI-*t* zeolites.** (a) TG analysis and (b) DTA curves.

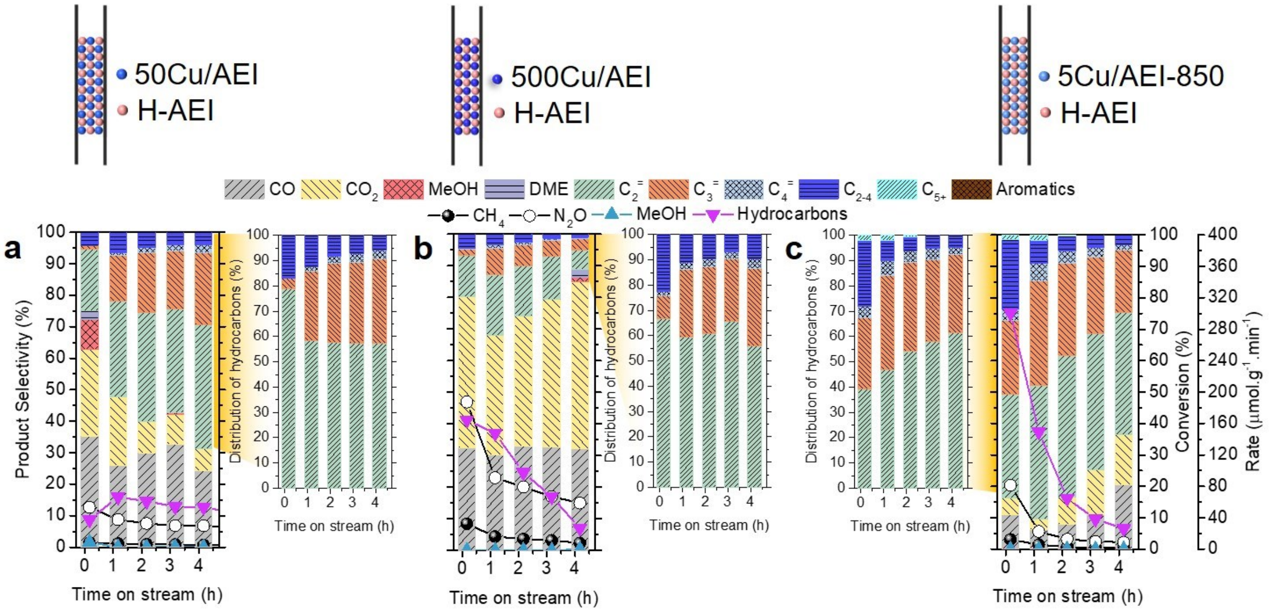


**Supplementary Figure 29. Effect of Cu component on the reaction performance of oxidation of methane by granule-stacking.** (a) 50 mg 50Cu/AEI and 50 mg H-AEI, (b) 50 mg 500Cu/AEI and 50 mg H-AEI, and (c) 50 mg 5Cu/AEI-850 and 50 mg H-AEI. Reaction conditions: 350 ºC, $CH_4/N_2O/H_2O/Ar$=10/10/2/3 ml·min$^{-1}$, WHSV=15000 ml·g$^{-1}$·h$^{-1}$.

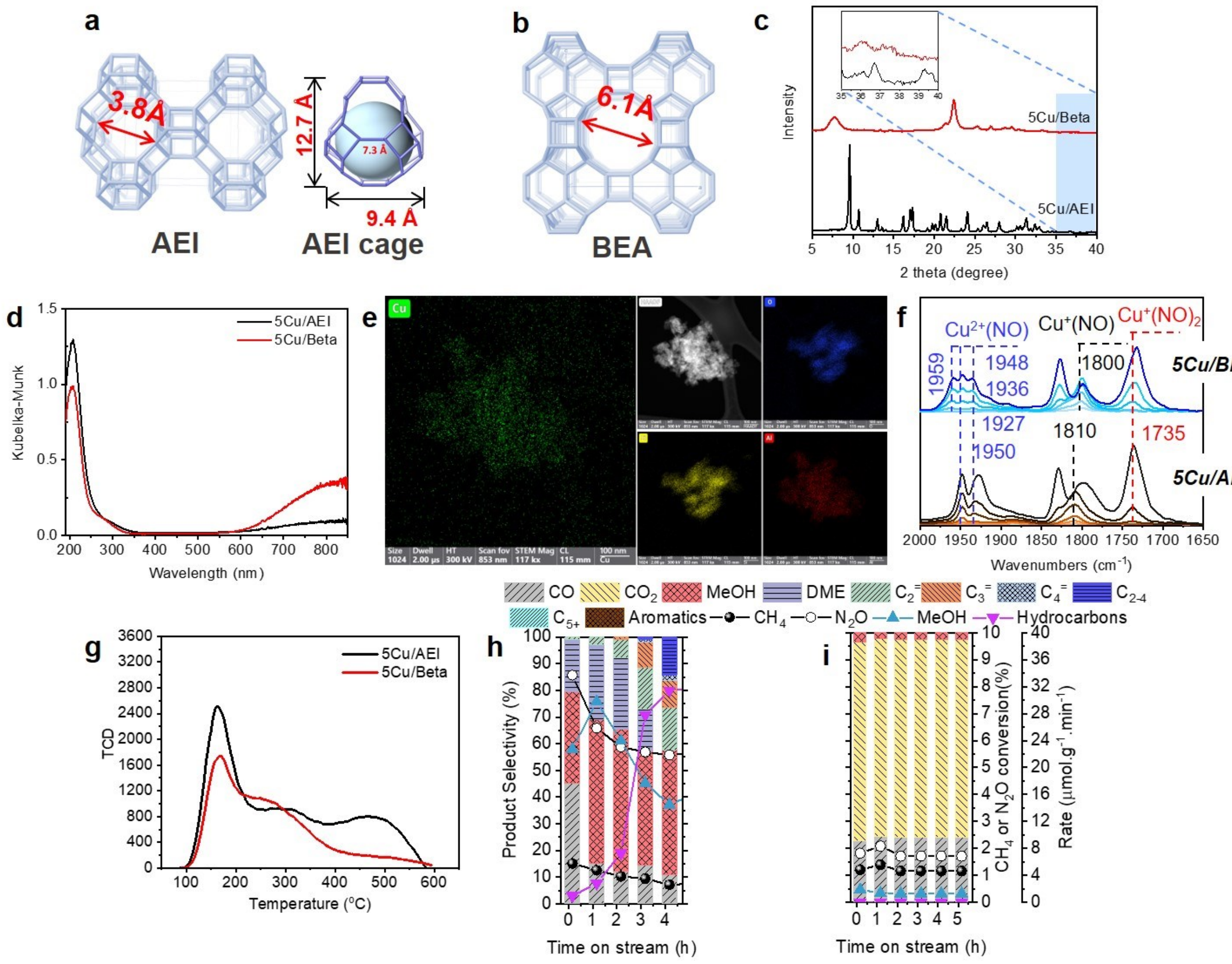

**Supplementary Figure 30. Effects of zeolite structure on reaction performance of direct oxidation of methane.** (a) Framework of AEI zeolite and the maximum diameter of a sphere that can diffuse along, and the AEI cage size that can included the maximum diameter. (b) Framework of BEA zeolite and the maximum diameter of a sphere that can diffuse along. (c) XRD patterns and (d) UV-vis spectra of the 5Cu/AEI and 5Cu/BEA zeolites. (e) HAADF-STEM images and EDS mapping images of 5Cu/BEA zeolite. (f) FTIR spectra of adsorbed NO (5-120 Pa) at -120 ºC of 5Cu/AEI and 5Cu/BEA zeolites. (g) $NH_3$-TPD curves of 5Cu/AEI and 5Cu/BEA zeolites. Reaction performance in the direct oxidation of methane for (h) 5Cu/AEI and (i) 5Cu/BEA. Reaction conditions: 100 mg catalyst, 350 ºC, $CH_4/N_2O/H_2O/Ar$=10/10/2/3 ml·min$^{-1}$, WHSV=15000 ml·g$^{-1}$·h$^{-1}$.

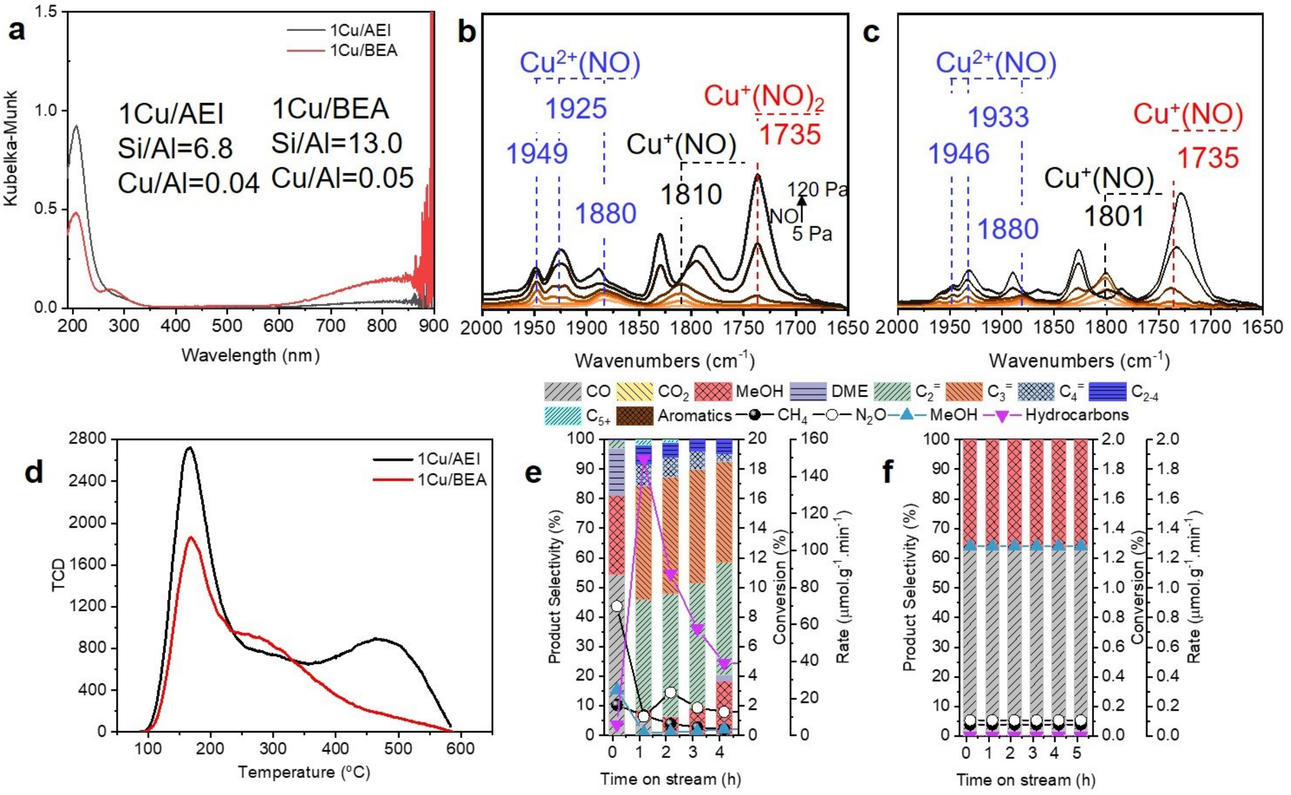

**Supplementary Figure 31. Effects of zeolite structure on reaction performance of direct oxidation of methane.** (a) Compare UV-vis spectra of the 1Cu/AEI and 1Cu/BEA zeolites. FTIR spectra of adsorbed NO (5-120 Pa) at -120 ºC of (b) 1Cu/AEI and (c) 1Cu/BEA zeolites. (d) Compare $NH_3$-TPD curves of 1Cu/AEI and 1Cu/BEA zeolites. Compare the reaction performance of (e) 1Cu/AEI and (f) 1Cu/BEA in methane oxidation reaction. Reaction conditions: 100 mg catalyst, 350 ºC, $CH_4/N_2O/H_2O/Ar$ = 10/10/2/3 ml·min$^{-1}$.

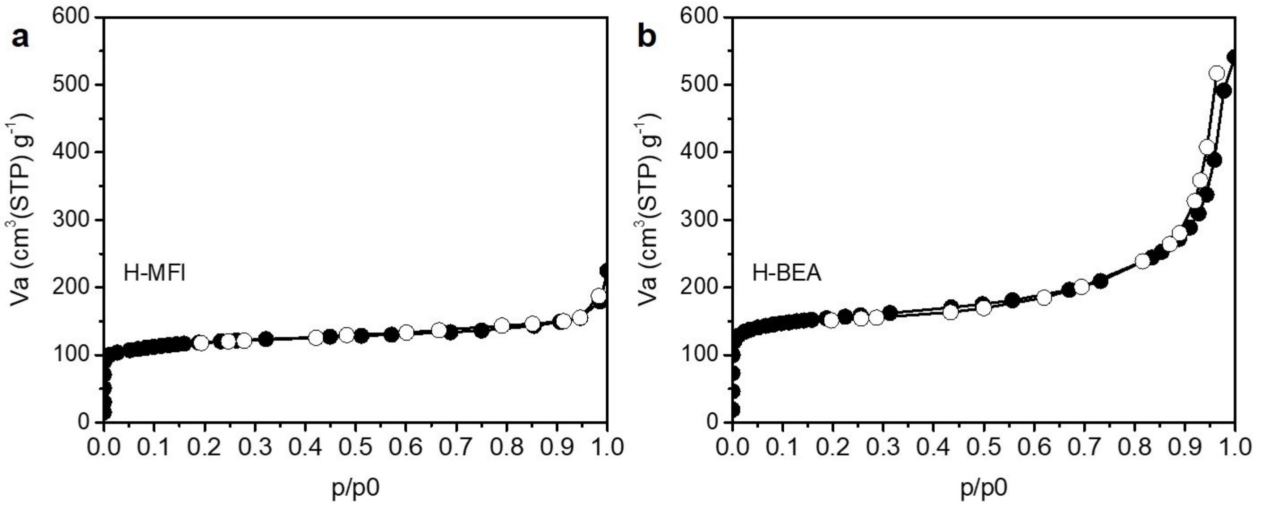


**Supplementary Figure 32. $N_2$ adsorption and desorption curves.** (a) H-MFI (JRC-Z5-30NH4) and (b) H-BEA (Zeolyst, CP814E*) zeolites.

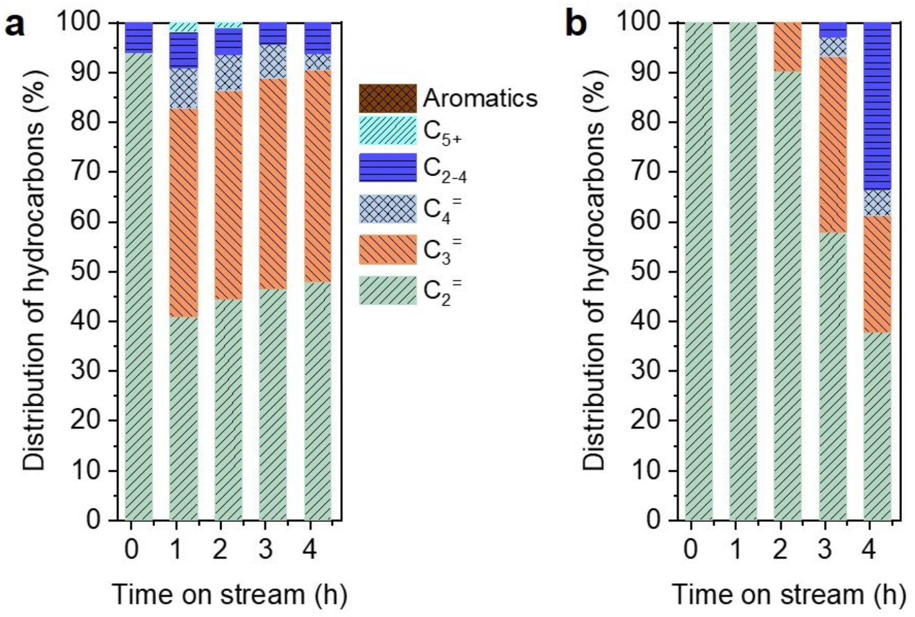


**Supplementary Figure 33. Distribution of hydrocarbons in direct oxidation of methane reaction.** (a) 1Cu/AEI and (b) 5Cu/AEI. Reaction conditions: 350 ºC, 100 mg catalyst, $CH_4/N_2O/H_2O/Ar$ = 10/10/2/3 ml·min$^{-1}$, WHSV=15000 ml·g$^{-1}$·h$^{-1}$.

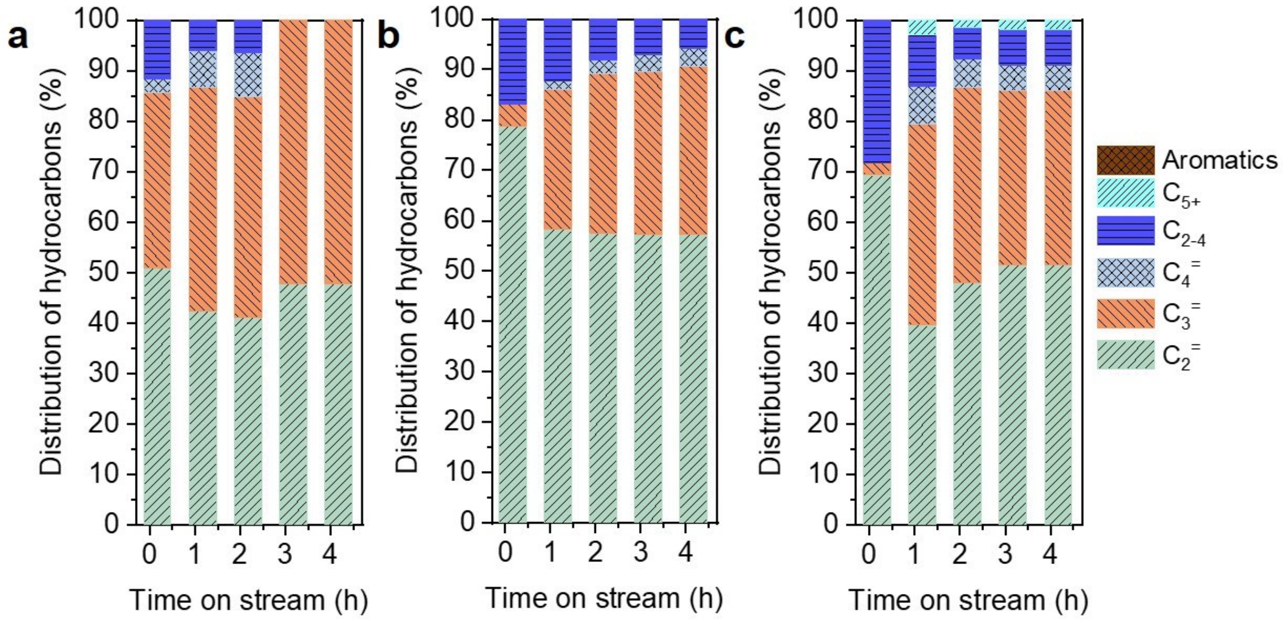


**Supplementary Figure 34. Distribution of hydrocarbons in direct oxidation of methane reaction.** (a) Dual bed (50 mg 50Cu/AEI+50 mg H-AEI); (b) granule-stacking of 50 mg 50Cu/AEI and 50 mg H-AEI with size of 400-600 μm; and (c) grinding-mixing 50% 50Cu/AEI and 50% H-AEI and then compressing to granules with size of 400-600 μm. Reaction conditions: total catalyst mass: 100 mg, 350 ºC, $CH_4/N_2O/H_2O/Ar$=10/10/2/3 ml·min$^{-1}$, WHSV=15000 ml·g$^{-1}$·h$^{-1}$.

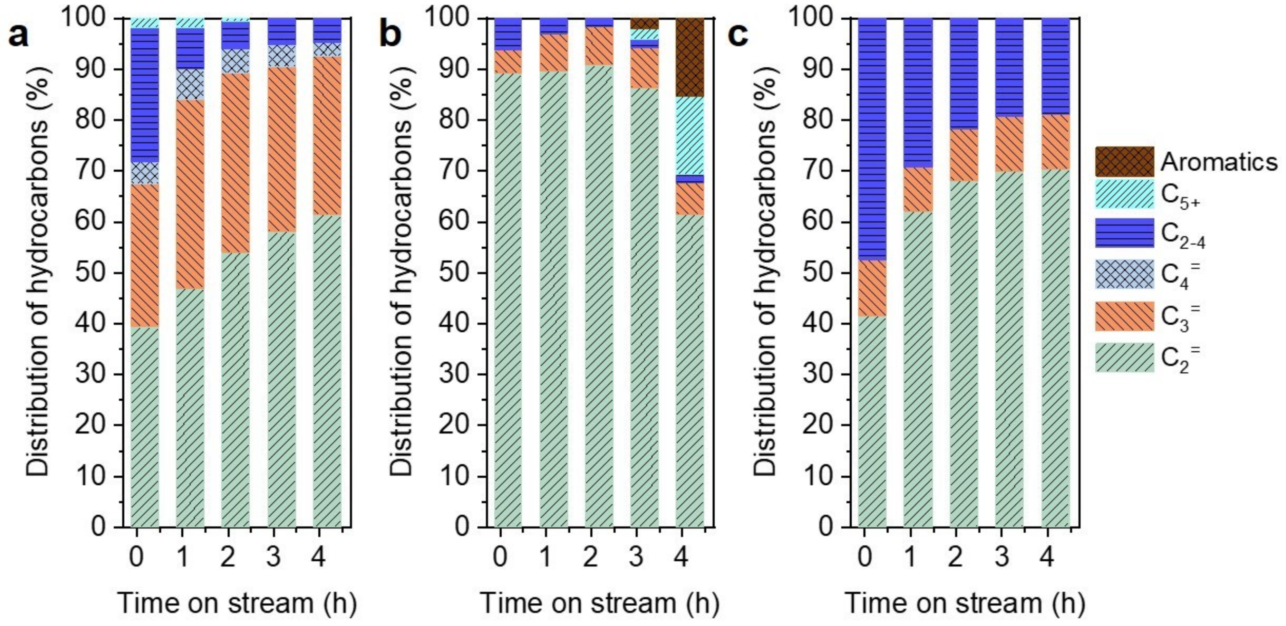


**Supplementary Figure 35. Distribution of hydrocarbons in direct oxidation of methane reaction.** 5Cu/AEI-850 granular stacking with (a) H-AEI, (b) H-MFI, (c) H-BEA in the direct oxidation of methane. Reaction conditions: 50 mg 5Cu/AEI-850 and 50 mg H-zeolite with the granule size of 400-600 μm, 350 ºC, $CH_4/N_2O/H_2O/Ar$=10/10/2/3 ml·min$^{-1}$, WHSV=15000 ml·g$^{-1}$·h$^{-1}$.

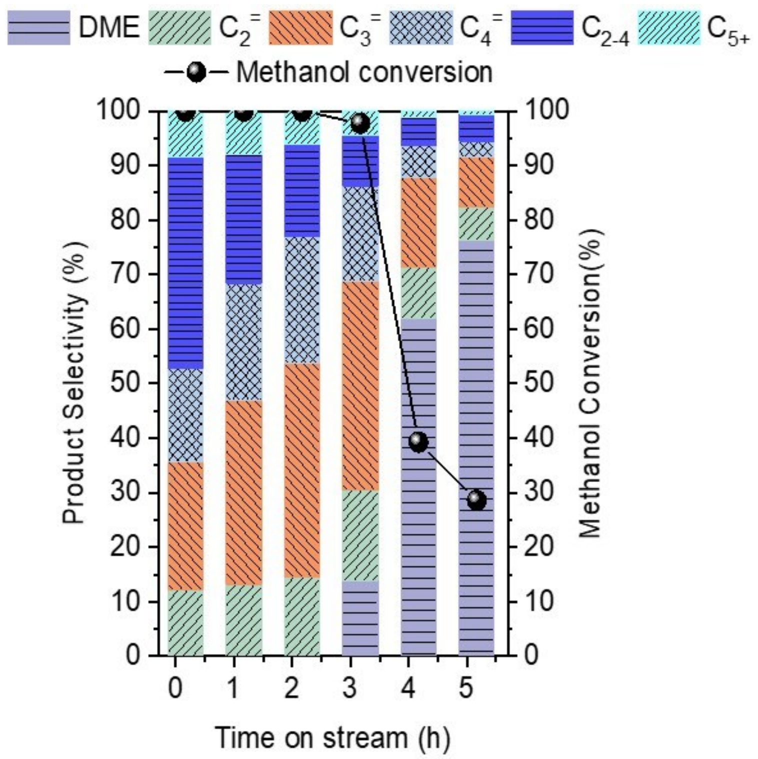


**Supplementary Figure 36. Time courses of methanol to hydrocarbon (MTH) reaction at 350 °C on H-AEI zeolite catalyst.** Reaction condition: 50 mg catalyst, $F_{MeOH}$ = 1 *u*l/min, $F_{He}$ = 11.6 ml/min, W/$F_{MeOH}$ = 33.7 g h/mol, $P_{methanol}$= 5 kPa.

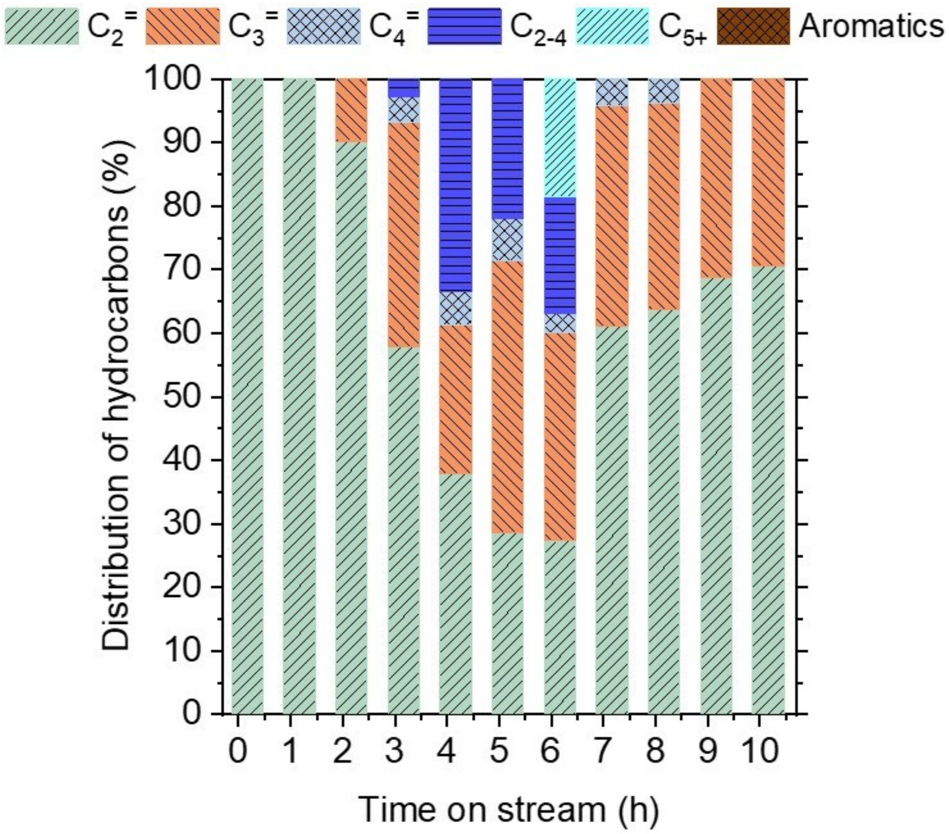


**Supplementary Figure 37. Hydrocarbons distribution of 5Cu/AEI-550 in the Figure 2a.** Reaction conditions: 350 ºC, 100 mg 5Cu/AEI-550 as catalyst, $CH_4/N_2O/H_2O/Ar$ = 10/10/2/3 ml·min$^{-1}$, WHSV=15000 ml·g$^{-1}$·h$^{-1}$.

**Supplementary Table 1. Chemical composition of zeolites.**

| Sample | Chemical Compositions [a] | | | Al content | | | Cu content | | | |
|---|---|---|---|---|---|---|---|---|---|---|
| | Si/Al | Cu/Al | Cu (wt.%) | Al/cage | Al /1000Å$^3$ | Al/unit cell | Cu/cage | Cu /1000Å$^3$ | Cu/unit cell | Cu/4 unit cells |
| H-AEI | 6.9 | - | - | 1.52 | 1.91 | 6.08 | 0 | 0 | 0 | 0 |
| 1Cu/AEI | 6.8 | 0.04 | 0.47 | 1.54 | 1.94 | 6.15 | 0.06 | 0.08 | 0.24 | 0.96 |
| 5Cu/AEI | 6.7 | 0.05 | 0.63 | 1.56 | 1.96 | 6.23 | 0.08 | 0.10 | 0.32 | 1.28 |
| 50Cu/AEI | 6.9 | 0.14 | 1.72 | 1.52 | 1.91 | 6.08 | 0.21 | 0.27 | 0.84 | 3.36 |
| 500Cu/AEI | 7.0 | 0.24 | 2.92 | 1.50 | 1.89 | 6.00 | 0.36 | 0.45 | 1.44 | 5.76 |
| 5Cu/BEA | 13.0 | 0.10 | 0.73 | - | 2.17 | 4.60 | - | 0.22 | 0.46 | 1.84 |
| 1Cu/BEA | 13.0 | 0.05 | 0.37 | - | 2.17 | 4.6 0 | - | 0.11 | 0.23 | 0.92 |

[a] by ICP-AES.

**Supplementary Table 2. Acid amount of zeolites measured by $NH_3$-TPD.**

| Sample | Equipment | Acid amount (mmol/g) [a] | | | |
|---|---|---|---|---|---|
| | | Weak | Medium | Strong | Total |
| H-AEI (BASF SE) | $NH_3$-TPD-B | 0.66 | 0.47 | 0.64 | 1.76 |
| 1Cu/AEI | | 0.57 | 0.57 | 0.58 | 1.73 |
| 5Cu/AEI | | 0.50 | 0.75 | 0.52 | 1.77 |
| 50Cu/AEI | | 0.44 | 0.96 | 0.50 | 1.91 |
| 500Cu/AEI | | 0.31 | 1.23 | 0.26 | 1.79 |
| 1Cu/AEI(spent) [b] | $NH_3$-TPD-A | 0.25 | 0.27 | 0.44 | 0.96 |
| 5Cu/AEI(spent) | | 0.26 | 0.46 | 0.37 | 1.09 |
| 50Cu/AEI(spent) | | 0.34 | 0.78 | 0.40 | 1.52 |
| 500Cu/AEI(spent) | | 0.27 | 0.97 | 0.25 | 1.49 |
| 1Cu/AEI-Re [c] | $NH_3$-TPD-A | 0.45 | 0.48 | 0.40 | 1.33 |
| 5Cu/AEI-Re | | 0.34 | 0.68 | 0.33 | 1.35 |
| 50Cu/AEI-Re | | 0.35 | 0.82 | 0.42 | 1.59 |
| 500Cu/AEI-Re | | 0.26 | 0.96 | 0.24 | 1.46 |
| 5Cu/AEI-750 | $NH_3$-TPD-B | 0.38 | 1.03 | 0.49 | 1.90 |
| 5Cu/AEI-850 | | 0.28 | 0.89 | 0.32 | 1.49 |
| 5Cu/BEA | | 0.26 | 0.58 | 0.27 | 1.11 |
| H-MFI (JRC-Z5-30$NH_4$) | | 0.53 | 0.30 | 0.64 | 1.47 |
| H-BEA (Zeolyst, CP814E*) | | 0.27 | 0.24 | 0.74 | 1.25 |
| 1Cu/BEA | | 0.31 | 0.36 | 0.43 | 1.10 |

[a] Determined by $NH_3$-TPD; fitting curves of the weak, medium, and strong acid amount were calculated at approximately 150, 300 and 400-450 °C, respectively. The samples of *x*Cu/AEI(spent) and *x*Cu/AEI-Re were measured by BELCAT-A with BELMass, the others samples were measured by BELCAT-B.

[b] *x*Cu/AEI(spent) represented the spent *x*Cu/AEI samples.

[c] *x*Cu/AEI-Re represented the regenerated *x*Cu/AEI by calcination at 550 °C for 5 h.

**Supplementary Table 3 Texture properties of zeolites measured by $N_2$ adsorption and desorption.**

| Sample | Textual Properties [a] | | |
|---|---|---|---|
| | $S_{BET}/m^2 \cdot g^{-1}$ [a] | $S_{EXT}/m^2 \cdot g^{-1}$ [b] | $V_{micro}/cm^3 \cdot g^{-1}$ [c] |
| H-AEI | 770 | 17 | 0.36 |
| 5Cu/AEI-550 | 735 | 18 | 0.36 |
| 5Cu/AEI-750 | 729 | 20 | 0.36 |
| 5Cu/AEI-850 | 727 | 22 | 0.37 |
| H-MFI | 443 | 46 | 0.17 |
| H-BEA | 583 | 257 | 0.11 |

[a] Calculated using the Brunauer–Emmett–Teller (BET) equation on the $N_2$ adsorption isotherms.

[b,c] Calculated by the t-plot method based on the adsorption isotherms.

**Supplementary References**

1. Parvulescu, A.-N. *et al.* AEI-type zeolitic material obtained from high temperature calcination and use as a catalyst. World Patent, WO2019/242615A1, 2019.

2. https://jrc.catsj.jp/sbform/sampledisplay.cgi

3. https://www.zeolyst.com/our-products/standard-zeolite-powders/zeolite-beta.html
